# Single-cell sensor analyses reveal signaling programs enabling Ras-G12C drug resistance

**DOI:** 10.1038/s41589-024-01684-4

**Published:** 2024-08-05

**Authors:** Jason Z. Zhang, Shao-En Ong, David Baker, Dustin J. Maly

**Affiliations:** 1 Department of Biochemistry, University of Washington, Seattle, WA USA; 2 Institute for Protein Design, University of Washington, Seattle, WA USA; 3 Howard Hughes Medical Institute, University of Washington, Seattle, WA USA; 4 Department of Pharmacology, University of Washington, Seattle, WA USA; 5 Department of Chemistry, University of Washington, Seattle, WA USA

**Keywords:** Cell signalling, Cancer, Chemical tools, Systems biology, Small molecules

## Abstract

Clinical resistance to rat sarcoma virus (Ras)-G12C inhibitors is a challenge. A subpopulation of cancer cells has been shown to undergo genomic and transcriptional alterations to facilitate drug resistance but the immediate adaptive effects on Ras signaling in response to these drugs at the single-cell level is not well understood. Here, we used Ras biosensors to profile the activity and signaling environment of endogenous Ras at the single-cell level. We found that a subpopulation of KRas-G12C cells treated with Ras-G12C-guanosine-diphosphate inhibitors underwent adaptive signaling and metabolic changes driven by wild-type Ras at the Golgi and mutant KRas at the mitochondria, respectively. Our Ras biosensors identified major vault protein as a mediator of Ras activation through its scaffolding of Ras signaling pathway components and metabolite channels. Overall, methods including ours that facilitate direct analysis on the single-cell level can report the adaptations that subpopulations of cells adopt in response to cancer therapies, thus providing insight into drug resistance.

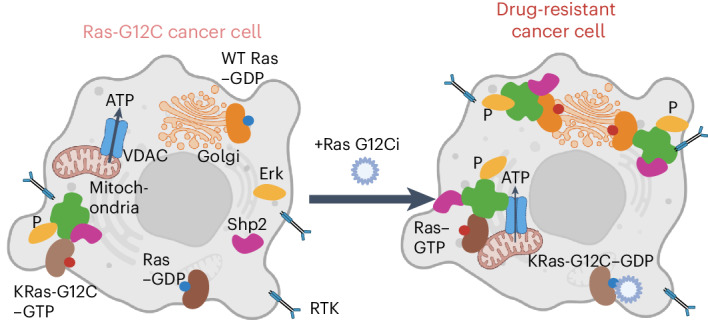

## Abstract (web summary)

Profiling endogenous Ras (rat sarcoma virus) activity during Ras-G12C inhibition at the single-cell level revealed signaling and metabolic changes driven by wild-type Ras at the Golgi and mutant KRas at the mitochondria and that major vault protein mediates these adaptations.

## Main

**Fig. 1:**
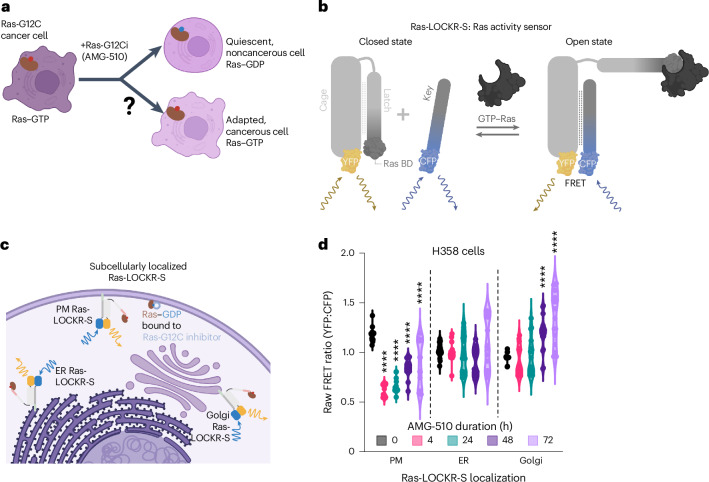
Ras-G12C–GDP inhibitor (AMG-510) treatment leads to Golgi-localized Ras activity. **a**, Schematic showing the question being studied: How does Ras-G12C inhibition lead to adaptive signaling and drug resistance? **b**, Schematic of Ras-LOCKR-S, a genetically encoded biosensor for measuring endogenous Ras activity. **c**, Schematic of localized Ras-LOCKR-S at three subcellular regions. **d**, Raw FRET ratios (YFP–CFP) of localized Ras-LOCKR-S in H358 cells treated for the times indicated with 100 nM AMG-510 (*n* = 17 cells per condition). PM, CV at 0 h = 5.7%, 4 h = 10%, 24 h = 11%, 48 h = 11% and 72 h = 26%; ER, CV at 0 h = 7%, 4 h = 9.4%, 24 h = 19%, 48 h = 15% and 72 h = 23%. Golgi, CV at 0 h = 4.6%, 4 h = 16%, 24 h = 19%, 48 h = 15% and 72 h = 19%. Statistical analysis was conducted using an ordinary two-way ANOVA. *P value comparison to the 0-h time point, from left to right: PM, 1.4 × 10^−7^, 3.5 × 10^−7^, 4.5 × 10^−6^ and 7.8 × 10^−5^; Golgi, 3.8 × 10^−5^ and 1.2 × 10^−5^. Source data

Mutations that overactivate the signaling enzyme Ras (rat sarcoma virus) are observed in one third of cancers^1^. Because of its strong oncogenic effect, there have been decades of effort to create therapeutics that target and inhibit mutant Ras. Covalent inhibitors against inactive Ras-G12C (bound to guanosine diphosphate (GDP))^2,3^ have recently demonstrated promising efficacy; however, most patients acquire drug resistance^4^ (Fig. 1a). Many reports have shown that resistance occurs through reactivation of the Ras–MAPK (mitogen-activated protein kinase) pathway^5–8^. However, it is unclear at the signaling level which cellular processes are affected by Ras inhibitors and what differentiates the drug-resistant cancer cell population from quiescent cells. KRas-G12C-expressing cancer cells also express wild-type (WT) HRas and NRas (H/NRas), thus WT Ras may have a compensatory role during mutant KRas inhibition^7^. While biochemically very similar, Ras isoforms are not functionally redundant, with one major difference being their subcellular localization. While all Ras isoforms can localize to the plasma membrane (PM), KRas4A also localizes to the mitochondrial outer membrane and H/NRas can dynamically shuttle to the endoplasmic reticulum (ER) and Golgi^9,10^. Thus, it is possible that KRas-G12C-driven cancer cells treated with Ras-G12C inhibitors adapt by reorganizing Ras signaling to enable drug resistance.

To understand the signaling mechanisms enabling drug resistance, we performed single-cell analyses of Ras signaling in response to Ras-G12C inhibition using our recently developed genetically encodable biosensors to measure the activity (Ras-LOCKR (latching orthogonal cage–key protein)-S) and environment (Ras-LOCKR-PL) of endogenous Ras^11^ (Fig. 1b). Both Ras-LOCKR-S and Ras-LOCKR-PL are two-protein-component biosensors that consist of a cage and key protein. The cage protein in LOCKR contains a ‘cage’ domain, a ‘latch’ domain that consists of binding elements that interact with the cage domain and the Ras–GTP (guanosine triphosphate)-binding domain (RasBD) from CRaf, as well as a portion of a split readout protein. The key protein contains a ‘key’ domain that can also interact with the cage domain of the cage protein and the other portion of the split readout protein. Without Ras–GTP, the latch domain of the cage is closed, with little interaction between the cage and key proteins, resulting in a low readout signal (‘closed state’). Ras–GTP binding to the RasBD within the latch domain decreases intramolecular interactions between the latch and cage domains, increases interactions between the cage and key proteins and brings the split readout proteins together (‘open state’). Ras-LOCKR-S uses Förster resonance energy transfer (FRET) efficiency between yellow (YFP) and cyan (CFP) fluorescent proteins to measure increased interaction between the cage and key proteins in the presence of Ras–GTP and Ras-LOCKR-PL uses the reconstitution of the split biotin ligase TurboID to promote localized biotinylation. These biosensors are tuned to be sensitive for endogenous active Ras, are compatible with live-cell imaging and can be localized to subcellular compartments (for example, organelles) to enable detection of Ras activity with subcellular resolution.

To determine whether WT Ras has a compensatory role during KRas-G12C inhibition, we used subcellularly localized Ras-LOCKR tools as a proxy to differentiate H/NRas from KRas because of the difference in localization of these Ras isoforms. Single-cell analysis of localized Ras-LOCKR-S demonstrated that a subpopulation of KRas-G12C-driven cancer cells treated with Ras-G12C–GDP inhibitors increased Golgi-localized WT H/NRas activity, which correlates with rebound MAPK signaling and drug-resistant cell growth. Application of Golgi-localized Ras-LOCKR-PL identified major vault protein (MVP) as a mediator of Golgi Ras activity through its interaction with several MAPK pathway components. Furthermore, we found that MVP is required for Ras-G12C–GDP inhibitor-promoted KRas4A activities at the mitochondria^12^. Recently developed Ras-G12C–GTP inhibitors may provide another avenue to tackle KRas-G12C-driven cancer cells^13^; however, we observed that Ras-G12C–GTP inhibition does not evade MVP-dependent WT Ras activation. Altogether, single-cell signaling analyses with our Ras-LOCKR tools enabled the discovery of MVP’s role in facilitating the resistance of a subpopulation of cancer cells to KRas-G12C inhibitors.

## Results

### AMG-510 treatment activates Ras at endomembranes

**Extended Data Fig. 1:**
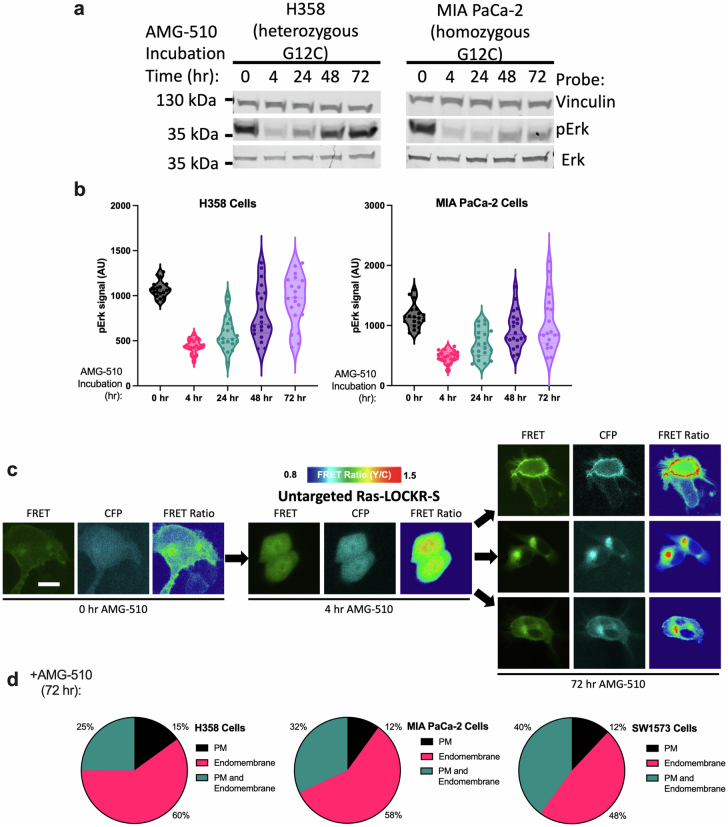
RasG12C-GDP inhibitor (AMG-510) treatment leads to rebound MAPK and endomembrane Ras signaling. (**a**) Representative immunoblot from four biological replicates of H358 (lung KRasG12C heterozygous) and MIA PaCa-2 (pancreas KRasG12C homozygous) cells treated for the times indicated with 100 nM AMG-510. (**b**) Violin plots of phospho-Erk (pErk) levels in H358 and MIA PaCa-2 cells treated with 100 nM AMG-510 for the times indicated. Each dot represents the average fluorescence intensity (from pErk immunostaining) of a single cell (n = 20 cells per condition). Coefficient of variation (CV) for H358 at 0 hour = 8.5%, 4 hour = 17%, 24 hour = 35%, 48 hour = 42%, 72 hour = 30%. CV for MIA PaCa-2 at 0 hour = 17%, 4 hour = 20%, 24 hour = 35%, 48 hour = 33%, 72 hour = 44%. Dashed line represents average and dotted lines represent 25^th^ and 75^th^ percentile. (**c**) Representative pseudocolor images of four biological replicates of H358 cells transfected with untargeted Ras-LOCKR-S treated with 100 nM AMG-510 over time. Scale bar = 10 *μ*m. (**d**) Pie charts showing the percentage of Ras-LOCKR-S-expressing KRasG12C cells (H358, MIA PaCa-2, SW1573) displaying FRET ratio increases either at PM and/or endomembranes after 72 hours of 100 nM AMG-510 treatment from four biological replicates (H358: n = 40 cells, MIA PaCa-2: n = 38 cells, SW1573: n = 36 cells). Cells were scored by visual inspection with PM signal indicating FRET ratio signal at cell periphery while endomembrane signal indicating FRET ratio signal concentrated in regions that are distinct from cell periphery. Source data

To understand the mechanisms of resistance against inhibitors targeting Ras-G12C–GDP such as AMG-510 (also known as sotorasib) (Fig. 1a), we used three KRas-G12C-expressing cell lines (H358 (heterozygous KRas-G12C), MIA PaCa-2 and SW1573 (homozygous KRas-G12C)) that show different MAPK signaling (measured by phosphorylated extracellular signal-regulated kinase (pErk) levels) rebound kinetics following AMG-510 treatment (Extended Data Fig. 1a). As Ras-G12C–GDP inhibition can lead to nonuniform changes across cells^4,5^, we probed whether rebound MAPK signaling downstream of Ras-G12C is also heterogeneous by measuring pErk levels at the single-cell level through immunostaining. In H358 cells, we observed that untreated cells had relatively uniform pErk levels (coefficient of variation (CV) = 8.5%) but highly heterogenous pErk levels were observed following prolonged AMG-510 treatment (CV = 30–42% (after 24–72 h of AMG-510 treatment)) (Extended Data Fig. 1b). Similarly, AMG-510 treatment led to heterogenous pErk levels in MIA PaCa-2 cells (Extended Data Fig. 1b). Thus, Ras-G12C–GDP inhibition leads to a subpopulation of cells with restored pErk levels, which we termed the ‘AMG-510-promoted signaling adaptive cell subpopulation’.

We next determined whether AMG-510 promotes heterogeneity in the activation state of Ras, which is upstream of Erk, by using Ras-LOCKR-S (see Main for details) to measure endogenous Ras activity (Ras–GTP) at the single-cell level (Fig. 1b). Using untargeted Ras-LOCKR-S to measure whole-cell Ras–GTP levels, we observed elevated FRET ratios (FRET channel divided by CFP channel) around the cell periphery in untreated cells and lower FRET ratios in the PM region 4 h after AMG-510 addition. Then, 72 h after AMG-510 addition, some cells displayed elevated FRET ratios at endomembranes, the PM or both regions simultaneously (Extended Data Fig. 1c,d), with a plurality of cells demonstrating elevated FRET ratios mainly at endomembranes. Because of the differential localization of the Ras isoforms, with mutant KRas-G12C mainly localized to the PM and WT H/NRas localized to endomembranes such as the ER and Golgi^14,15^, we reasoned that endomembrane Ras activity can serve as an indicator for WT H/NRas activity.

**Extended Data Fig. 2:**
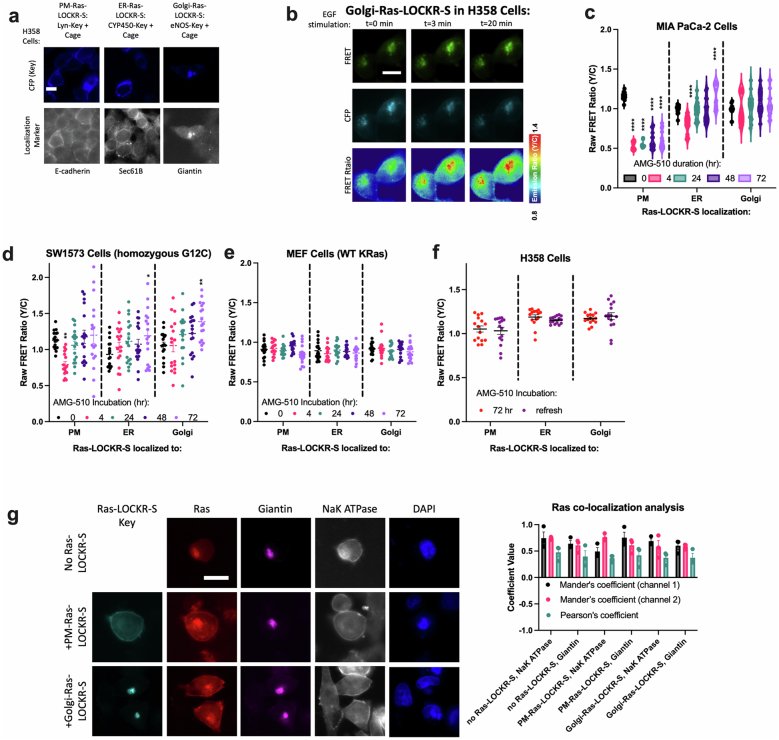
AMG-510 treatment leads to Ras activation at the golgi. (**a**) Representative epifluorescence images from three biological replicates of H358 cells transfected with subcellularly localized Ras-LOCKR-S and immunostained for respective localization markers. Localization sequences: PM = N-terminus of Lyn, ER = N-terminus of CYP450, Golgi=N-terminus of eNOS. Scale bar = 10 *μ*m. (**b**) Representative images from three biological replicates of H358 cells transfected with golgi-Ras-LOCKR-S and stimulated with 100 ng/mL EGF. Shown are live cell epifluorescence images. YFP/CFP FRET channel on top, CFP channel on bottom, and pseudocolored FRET ratios on the bottom. Scale bar = 10 μm. (**c–e**) Raw FRET ratios of localized Ras-LOCKR-S in MIA PaCa-2 (**c**), SW1573 (**d**), or WT MEF (**e**) cells treated over time with 100 nM AMG-510 (n = 17 cells per condition). For MIA PaCa-2 data (**c**): PM: CV at 0 hour = 4.1%, 4 hour = 9.4%, 24 hour = 6.4%, 48 hour = 18%, 72 hour = 19%. ER: CV at 0 hour = 5.2%, 4 hour = 14%, 24 hour = 12%, 48 hour = 15%, 72 hour = 13%. Golgi: CV at 0 hour = 7.4%, 4 hour = 18%, 24 hour = 16%, 48 hour = 14%, 72 hour = 16%. Statistics: Ordinary two-way ANOVA. *p-values are comparing to 0 hour AMG-510 time point: (**c**) PM: 3.2 × 10^−6^, 5.4 × 10^−6^, 4.1 × 10^−5^, 2.1 × 10^−5^, ER: 9.6 × 10^−6^, 5.4 × 10^−6^, (**d**) PM: 3.4 × 10^−3^, ER: 0.019, Golgi: 6.7 × 10^−3^. (**f**) Raw FRET ratios of localized Ras-LOCKR-S in H358 cells treated with 100 nM AMG-510 for 72 hours and then refreshed with 100 nM AMG-510 for another 2 hours (n = 15 cells per condition). (**g**) H358 cells either with no transfection, transfected with PM-Ras-LOCKR-S, or transfected with golgi-Ras-LOCKR-S were immunostained for Ras, giantin (golgi marker), and NaK ATPase (PM marker). Left: Representative epifluorescence images of fixed cells from three biological replicates. Right: Co-localization analysis measuring co-localization of endogenous Ras with the golgi and PM markers (n = 3 experiments). Scale bar = 10 μm. Source data

To further explore the observed endomembrane Ras activity promoted by AMG-510, we used subcellularly localized Ras-LOCKR-S constructs that contain N-terminal localization sequences fused to the key of Ras-LOCKR-S (Fig. 1c and Extended Data Fig. 2a,b). In KRas-G12C cells, AMG-510 addition led to rapid (4 h) decreases followed by a gradual, partial rebound in PM Ras-LOCKR-S FRET ratios (Fig. 1d and Extended Data Fig. 2c,d). In contrast, AMG-510 treatment led to increased levels of Ras activation at endomembranes. H358 cells, which showed significant rebound in pErk levels (Extended Data Fig. 1a), displayed no significant changes in ER Ras activity but robust increases in Golgi Ras-LOCKR-S FRET ratios (Fig. 1d). Similar to the nonuniform pErk levels we observed (Extended Data Fig. 1b), prolonged AMG-510 treatment resulted in heterogenous increases (CV at 72 h = 19–23%) in Ras–GTP levels at endomembranes in H358 cells. SW1573 and MIA PaCa-2 cells showed FRET ratio increases with both ER Ras-LOCKR-S and Golgi Ras-LOCKR-S (Extended Data Fig. 2c,d). Meanwhile, WT mouse embryonic fibroblasts (MEFs) showed no FRET ratio changes during AMG-510 treatment (Extended Data Fig. 2e) and refresh of AMG-510 after 72 h treatment had a minimal effect on localized Ras activities (Extended Data Fig. 2f). Immunostaining of H358 cells demonstrated that there is a pool of endogenous Ras that is localized to the Golgi and that expression of our localized Ras-LOCKR-S constructs do not seem to perturb overall Ras localization (Extended Data Fig. 2g). Altogether, these results demonstrate that AMG-510 treatment leads to Ras activation at endomembranes in a subpopulation of cells.

### AMG-510 resistance through WT active Ras at the Golgi

**Extended Data Fig. 3:**
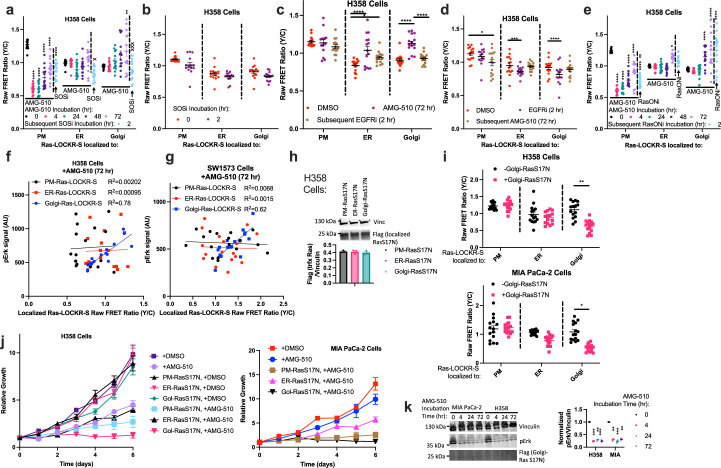
Effects of AMG-510-promoted golgi-localized Ras activity. (**a-e**) Raw FRET ratios (Y/C) of H358 cells expressing localized Ras-LOCKR-S that were treated either (**a**) with 100 nM AMG-510 followed by 1μM Sos inhibitor (SOSi) BI-3406 (n = 17 cells per condition), (**b**) with 1μM Sos inhibitor BI-3406 (n = 14 cells per condition), (**c**) with 100 nM AMG-510 followed by 50μg/mL Cetuximab (EGFRi) (n = 17 cells per condition), (**d**) with 50μg/mL Cetuximab followed by 100 nM AMG-510 (n = 15 cells per condition), or (**e**) with 100 nM AMG-510 followed by 100 nM RasG12C-GTP (RasON) inhibitor (RasONi) RMC-6291 (**e**) (n = 14 cells per condition). Statistics: Ordinary two-way ANOVA. *p-values are comparison to 0 hour time point or as indicated, left to right: (**a**) PM: 5.1 × 10^−8^, 4.5 × 10^−8^, 2.5 × 10^−6^, 3.5 × 10^−6^, 9 × 10^−7^, Golgi: 8.7 × 10^−3^, (**c**) ER: 7.5 × 10^−6^, 0.031, Golgi: 4.3 × 10^−6^, 3.2 × 10^−6^, (**d**) PM: 0.035, ER: 1.5 × 10^−4^, Golgi: 8.1 × 10^−5^, (**e**) PM: 4.1 × 10^−7^, 9.7 × 10^−7^, 1.5 × 10^−6^, 8.3 × 10^−7^, 1.3 × 10^−5^, ER: 0.011, Golgi: 1.7 × 10^−6^, 9.1 × 10^−8^, 5.5 × 10^−5^. x is p-value comparing to 72 hour time point, left to right: (**a**) ER: 0.029, Golgi: 1.5 × 10^−4^, (**e**) PM: 7.7 × 10^−3^. (**f,g**) Scatterplot comparing localized Ras-LOCKR-S raw FRET ratio to pErk immunostaining fluorescence in H358 (**f**) or SW1573 (**g**) cells treated with 100 nM AMG-510. Each dot represents a single cell where cells were transfected, treated with AMG-510, fixed, immunostained with pErk antibody, and imaged (n = 17 cells per condition). (**h**) Top: Representative immunoblot of H358 cells stably expressing localized RasS17N (see Methods) (n = 3 experiments). Bottom: Densitometry analysis. (**i**) Raw FRET ratios of H358 or MIA PaCa-2 cells with or without stable expression of Golgi-RasS17N and transfected with localized Ras-LOCKR-S (n = 15 cells per condition). Imaging was 24 hours after transfection. Statistics: Ordinary two-way ANOVA. *p-value, top to bottom: 7.7 × 10^−3^, 0.029. (**j**) Cell growth curves of H358 (left) or MIA PaCa-2 (right) cells with or without stable expression of localized RasS17N and treatment with 100 nM AMG-510 or DMSO (n = 3 experiments). (**k**) Left: Representative immunoblot of MIA PaCa-2 or H358 cells stably expressing localized RasS17N and treated with 100 nM AMG-510 (n = 4 experiments). Right: Densitometry analysis. Data normalized to 0 hour time point. Statistics: Ordinary two-way ANOVA. *p-values are comparison to 0 hour time point, left to right: H358: 4 × 10^−4^, 3.5 × 10^−3^, 8.6 × 10^−4^, MIA PaCa-2: 3 × 10^−3^, 9.7 × 10^−4^, 6.4 × 10^−3^. For all, bars or lines represent average and error bars represent s.e.m. Source data

To test whether WT Ras has a role in redistributed Ras signaling in response to Ras-G12C inhibition, we measured how subcellular Ras activation levels respond to inhibition of Sos1 guanine exchange factors (GEFs) and epidermal growth factor receptor (EGFR) because WT Ras shows a greater dependence on GEF and EGFR activity than Ras-G12C. H358 cells expressing subcellularly localized Ras-LOCKR-S were treated with AMG-510 for 72 h followed by treatment with the Sos1 inhibitor BI-3406 (SOSi, BI-3406)^16^ or EGFR inhibitor (EGFRi) cetuximab^17^ for 2 h (Extended Data Fig. 3a–c). Both SOSi and EGFRi treatment significantly decreased AMG-510-promoted Golgi Ras activation but minimally affected Ras–GTP levels at the PM. SOSi alone minimally affected Ras activity at all of the tested subcellular locations (Extended Data Fig. 3b) and EGFRi alone decreased Ras activity at the ER and Golgi and prevented AMG-510-promoted Ras activation at the ER and Golgi (Extended Data Fig. 3d). These results suggest that the observed Ras activities at the ER and Golgi are from WT Ras. Further supporting this notion, we found that treating cells with an inhibitor that targets GTP-bound Ras-G12C (RasONi, RMC-6291)^13^ 72 h after adding AMG-510 significantly decreased rebound Ras activity at the PM but did not diminish Golgi Ras activation (Extended Data Fig. 3e). Altogether, these results indicate that AMG-510 treatment leads to WT Ras activation at endomembranes and KRas-G12C reactivation at the PM, possibly through Ras-G12C overexpression as a drug adaptation^5^.

**Extended Data Fig. 4:**
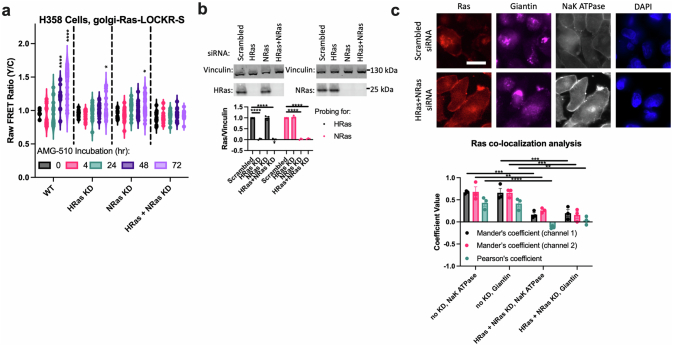
AMG-510-mediated golgi-localized Ras activity is through WT H/NRas. (**a**) Raw FRET ratios (Y/C) of golgi-Ras-LOCKR-S in H358 cells transfected with HRas and/or NRas siRNA and 2 days later subsequently treated for the indicated times with 100 nM AMG-510 (n = 17 cells per condition). Statistics: Ordinary two-way ANOVA. *p-value is comparison to 0 hour AMG-510 time point, from left to right: WT: 5.7 × 10^−6^, 8.1 × 10^−8^, HRas KD: 0.021, NRas KD: 0.027. (**b**) Top: Representative immunoblot of H358 cells transfected with scrambled, HRas, and/or NRas siRNA (n = 3 experiments). Bottom: Densitometry analysis with bars representing average and error bars representing s.e.m. for all three siRNA replicates. Data are normalized to scrambled siRNA transfection. Statistics: Ordinary two-way ANOVA. *p-values are comparison to scrambled siRNA, from left to right: 2.9 × 10^−7^, 5.4 × 10^−6^, 8.7 × 10^−7^, 4.3 × 10^−6^. (**c**) Top: Representative epifluorescence images of H358 cells transfected with scrambled or HRas + NRas siRNA and 2 days later subsequently immunostained for Ras, giantin (golgi marker), and NaK ATPase (PM marker) (n = 3 experiments). Bottom: Co-localization analysis measuring co-localization of endogenous Ras with the golgi and PM markers (n = 3 experiments). Scale bar = 10 μm. Statistics: Ordinary two-way ANOVA. *p-values, from left to right: 3.4 × 10^−4^, 8.8 × 10^−3^, 8.8 × 10^−8^, 8.7 × 10^−4^, 7.5 × 10^−4^, 2.3 × 10^−3^. Bars represent average from three biological replicates and error bars represent s.e.m. Source data

While each of the three subcellular regions tested (PM, ER and Golgi) showed dynamic changes in Ras activities after AMG-510 treatment, we wondered which subcellular pool of active Ras is most relevant to the AMG-510-promoted signaling adaptive cell subpopulation. At the single-cell level, only Golgi Ras activity (measured by Golgi Ras-LOCKR-S FRET ratios) correlated (*R*^2^ = 0.78, H358 cells) with Erk activation (pErk immunostaining) 72 h after AMG-510 treatment in the KRas-G12C-driven cancer cell lines that were tested (Extended Data Fig. 3f,g). Consistent with Ras activity at the Golgi having a functional role in AMG-510-resistant cell growth, only Golgi-localized dominant negative Ras mutant (Ras-S17N)^18^, which decreased Ras activity at the Golgi but not the ER or PM (Extended Data Fig. 3h,i), prevented cell proliferation in H358 cells after AMG-510 treatment (Extended Data Fig. 3j). Golgi-localized Ras-S17N also prevented global pErk rebound after 72 h of AMG-510 treatment (Extended Data Fig. 3k). To further probe which Ras isoform is responsible for this AMG-510-promoted Golgi Ras activation, H/NRas were knocked down by small interfering RNA (siRNA), which abrogated AMG-510-promoted Golgi Ras activation (Extended Data Fig. 4a,b) and led to lower endomembrane Ras localization (Extended Data Fig. 4c). Altogether, we demonstrate that Golgi-localized WT H/NRas–GTP enables rebound MAPK signaling and oncogenic cell growth in the AMG-510-promoted signaling adaptive cell subpopulation.

### AMG-510-promoted signaling requires MVP

**Fig. 2:**
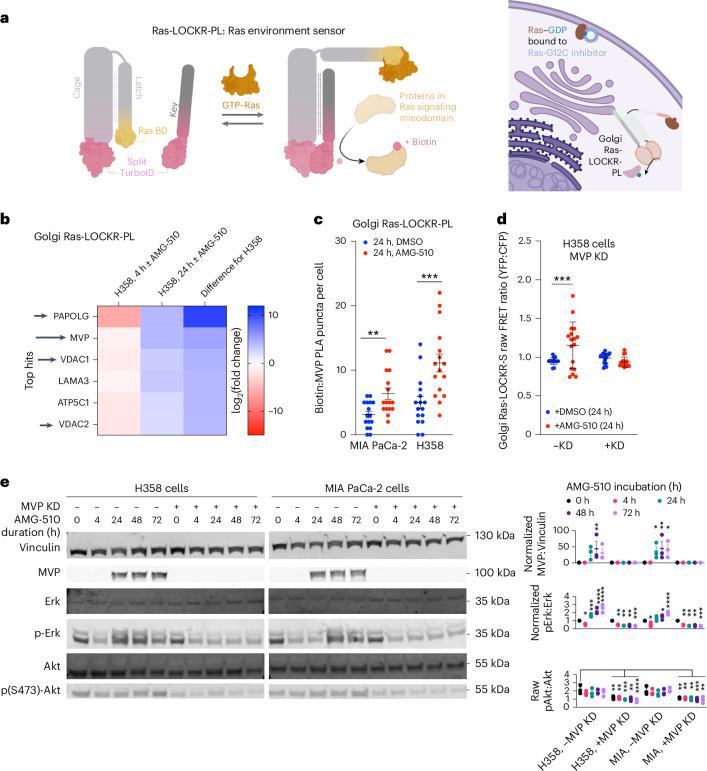
MVP contributes to AMG-510-promoted Ras signaling at the Golgi. **a**, Left, schematic of Ras-LOCKR-PL, a Ras activity-dependent proximity labeler. Right, schematic of Golgi-localized Ras-LOCKR-PL in action. **b**, H358 cells expressing Golgi-localized Ras-LOCKR-PL were cotreated with 500 μM biotin and 100 nM AMG-510 or DMSO followed by lysis and enrichment with streptavidin beads. Streptavidin-enriched proteins were then trypsinized and subjected to LC–MS analysis (*n* = 4 experimental repeats). The values shown in the heat map are the log_2_ ratio of protein intensities between cells treated with DMSO and those treated with AMG-510 for 4 h (column 1) and between cells treated with DMSO and those treated with AMG-510 for 24 h (column 2). The third column is the difference between columns 1 and 2. Details are in Methods. Black arrows are MS hits that were tested further. **c**, Number of PLA puncta (anti-biotin and anti-MVP) per cell. MIA PaCa-2 or H358 cells were transfected with Golgi-localized Ras-LOCKR-PL, incubated with 500 μM biotin and DMSO or 100 nM AMG-510 and then analyzed with PLA (*n* = 16 cells). Each dot represents a single cell. Bars show the mean and error bars represent the s.e.m. Statistical analysis was conducted using an ordinary two-way ANOVA. *P values, from left to right: 8.4 × 10^−3^ and 4.5 × 10^−4^. **d**, Comparison of raw FRET ratios in H358 cells transfected with Golgi Ras-LOCKR-S and either MVP or scrambled siRNA and then treatment with DMSO or 100 nM AMG-510 (*n* = 17 cells per condition). Statistical analysis was conducted using an ordinary two-way ANOVA. *P* = 3.0 × 10^−4^. **e**, Left, representative immunoblot from three biological replicates of H358 and MIA PaCa-2 with or without MVP siRNA transfection before and after treatment with 100 nM AMG-510. Right, densitometry quantification of immunoblots either not normalized (pAkt versus Akt) or normalized to 0 h (the remainder) of three replicates. The middle line represents the average and error bars represent the s.e.m. Statistical analysis was conducted using an ordinary one-way ANOVA; comparisons are as indicated or to the 0-h AMG-510 time point. *P values, from left to right: MVP versus Vinculin, 5.7 × 10^−3^, 0.024, 7.1 × 10^−3^ and 0.027; pErk versus Erk, 0.031, 8.1 × 10^−4^, 9.1 × 10^−5^, 8.7 × 10^−5^, 4.1 × 10^−3^, 5.3 ×;10^−3^, 4.2 × 10^−3^, 8.2 × 10^−4^, 0.033, 0.029, 9.8 × 10^−5^, 5.1 × 10^−3^, 5.7 × 10^−3^, 2.1 × 10^−3^ and 8.1 × 10^−4^; pAkt versus Akt, 4.5 × 10^−3^, 5.4 × 10^−3^, 7.7 × 10^−4^, 5.1 × 10^−3^, 7.5 × 10^−5^, 3.3 × 10^−3^, 4.1 × 10^−3^, 5.1 × 10^−3^, 4.1 × 10^−4^ and 7.0 × 10^−3^. Source data

**Extended Data Fig. 5:**
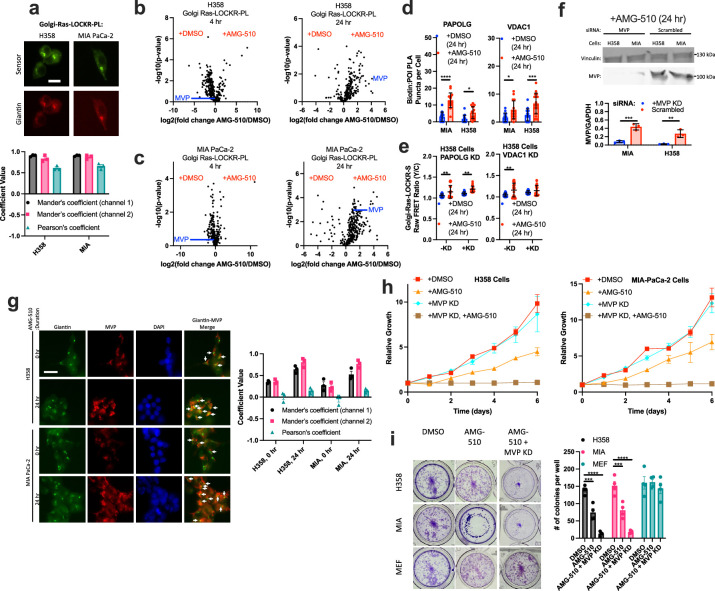
MVP facilitates AMG-510-dependent oncogenic signaling and cell growth. (**a**) Top: Representative images from three biological replicates of H358 or MIA PaCa-2 cells transfected with golgi-localized Ras-LOCKR-PL and immunostained for giantin. Bottom: Co-localization analysis between golgi-Ras-LOCKR-PL Key and giantin (n = 3 experiments). Scale bar = 10 μm. (**b,c**) Volcano plots of MS results of H358 (b) or MIA PaCa-2 (c) cells expressing localized Ras-LOCKR-PL and incubated for 24 hours with 500 μM biotin and either with 100 nM AMG-510 or DMSO. X-axis is log_2_ratio of protein intensities between cells treated with AMG-510 versus DMSO for 4 hours or for 24 hours. Y-axis is student’s two-way t-test p-value (see Methods). (**d**) MIA PaCa-2 or H358 cells were transfected with golgi-localized Ras-LOCKR-PL, incubated with 500 μM biotin and either with DMSO or 100 nM AMG-510, and underwent PLA (n = 16 cells). The number of PLA puncta per cell are shown. Each dot represents a single cell. Statistics: Student’s two-way t-test. *p-values, left to right: 8.7 × 10^−8^,, 0.033, 0.02, 5.1 × 10^−4^. (**e**) Raw FRET ratios of H358 cells transfected with either no siRNA or MVP siRNA and then Ras-LOCKR-S, followed by treatment with 100 nM AMG-510 or DMSO for 24 hours (n = 17 cells per condition). Statistics: Student’s two-way t-test. *p-values, left to right: 7.1 × 10^−3^, 5.9 × 10^−3^, 6.9 × 10^−3^. (**f**) Representative immunoblot of MIA PaCa-2 or H358 cells transfected with MVP or scrambled siRNA for 2 days and subsequently treated with 100 nM AMG-510 for 24 hours (n = 3 experiments). Statistics: Student’s two-way t-test. *p-values, left to right: 7.1 × 10^−4^, 1.5 × 10^−3^. (**g**) Left: Representative images of H358 and MIA PaCa-2 cells treated without or with 100 nM AMG-510 and immunostained for MVP and giantin. Right: Colocalization analysis between giantin and MVP (n = 3 experiments). Scale bar = 10 μm. (**h**) Cell growth curves of H358 (left) or MIA PaCa-2 (right) cells transfected either with no siRNA or MVP siRNA followed by treatment with 100 nM AMG-510 or DMSO (n = 3 experiments). (**i**) Representative images of colony formation assay of H358, MIA PaCa-2, or MEF transfected either with no siRNA or MVP siRNA followed by treatment either with DMSO or 100 nM AMG-510 (n = 3 experiments). Statistics: Ordinary two-way ANOVA. *p-values, left to right: 5.1 × 10^−4^, 2.2 × 10^−7^, 8.7 × 10^−4^, 8.5 × 10^−6^. For all, bars or lines represent average and error bars represent s.e.m. Source data

**Extended Data Fig. 6:**
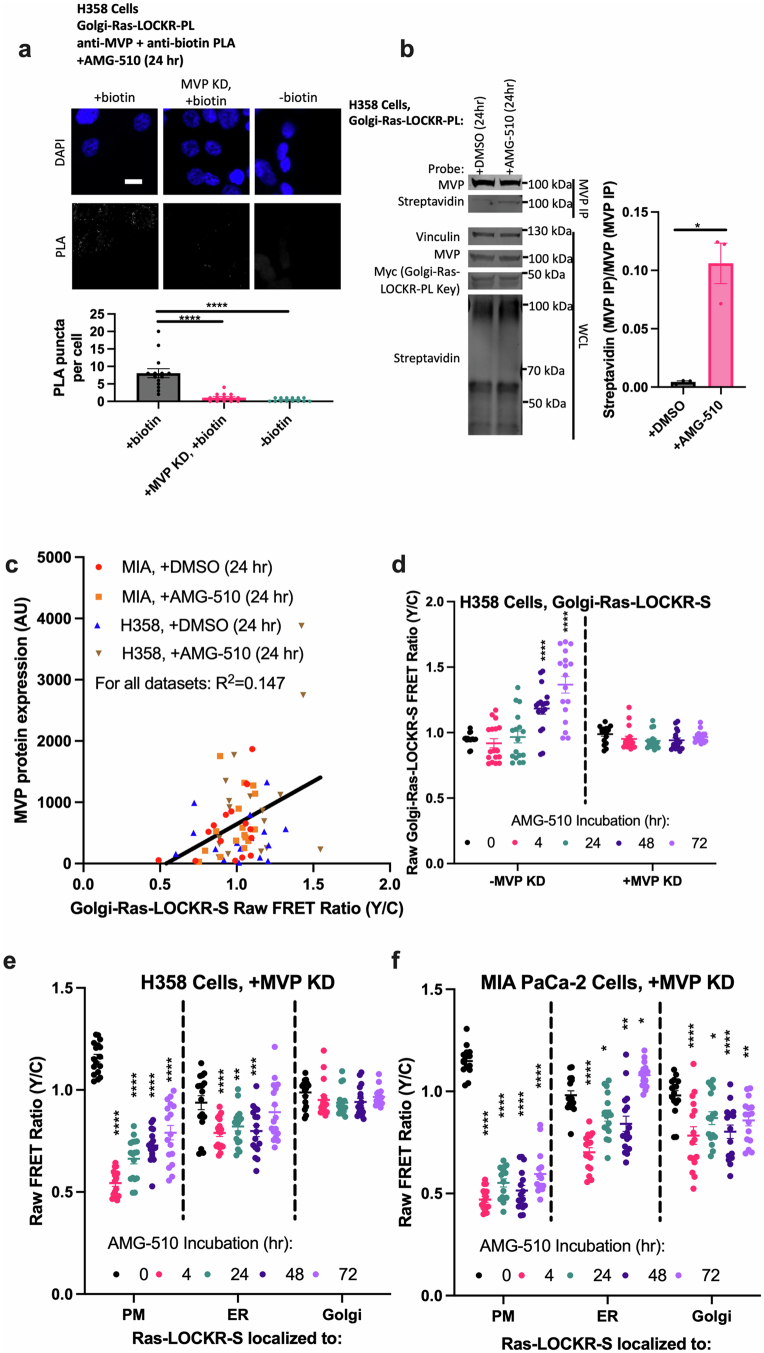
MVP at the golgi-Ras signalosome typifies the AMG-510-promoted signaling adaptive cell subpopulation. (**a**) Top: Representative epifluorescence images from three biological replicates of H358 transfected with either no or MVP siRNA and then golgi-localized Ras-LOCKR-PL, treated with 100 nM AMG-510 for 1 day, and either treated also with 500 μM biotin, treated either with 500 μM biotin or DMSO. Cells then were fixed and subjected to PLA using anti-MVP and anti-biotin. Bottom: Bar graph quantifying number of PLA puncta per condition (n = 14 cells). Scale bar = 10 μm. Statistics: Student’s two-way t-test. *p-values, left to right: 7.1 × 10^−7^, 6.1 × 10^−6^. (**b**) Left: Representative immunoblot of three biological replicates of H358 cells transfected with golgi-Ras-LOCKR-PL, treated with either DMSO or 100 nM AMG-510 for 24 hours, and then subjected to MVP pull down. Right: Bar graph quantifying the amount of biotinylated MVP over total amount of MVP (n = 3 experiments). Statistics: Student’s two-way t-test. *p-value: 0.011. (**c**) Scatterplot comparing MVP expression by immunostaining (y-axis) to golgi-Ras-LOCKR-S raw FRET ratios (x-axis). MIA PaCa-2 or H358 cells were transfected with golgi-Ras-LOCKR-S, treated either with DMSO or 100 nM AMG-510 for 24 hours, immunostained, and then imaged. Each dot represents a single cell where both measurements were done in fixed cells (n = 16 cells per condition, 64 cells in total). (**d**) Raw FRET ratios in H358 cells transfected either with scrambled or MVP siRNA and then golgi-Ras-LOCKR-S, followed by subsequent treatment over time with 100 nM AMG-510 (n = 17 cells per condition). The 0 and 72 hour dataset is also shown in Fig. 3d. Statistics: Ordinary two-way ANOVA, comparison to 0 hour AMG-510 time point. *p-values are comparison to 0 hour time point, left to right: 3.3 × 10^−6^, 4.1 × 10^−6^. (**e,f**) Raw FRET ratios of subcellularly localized Ras-LOCKR-S in either H358 (**e**) or MIA PaCa-2 (**f**) cells transfected MVP siRNA prior to Ras-LOCKR-S transfection, followed by treatment over time with 100 nM AMG-510 (n = 17 cells per condition). The H358 golgi datasets are the same as shown in Fig. 3d and Extended Data Fig. 6d. Statistics: Ordinary two-way ANOVA. *p-values are comparison to 0 hour point, left to right: (**e**) PM: 5.1 × 10^−6^, 3.3 × 10^−7^, 8.1 × 10^−6^, 5.6 × 10^−5^, ER: 8.1 × 10^−8^, 5.1 × 10^−3^, 7.7 × 10^−4^, (**f**) PM: 5.6 × 10^−7^, 5.6 × 10^−6^, 7.1 × 10^−7^, 8.1 × 10^−5^, ER: 1.5 × 10^−6^, 0.018, 4.4 × 10^−3^, 0.019, Golgi: 6.1 × 10^−5^, 0.012, 7.3 × 10^−5^, 5.3 × 10^−3^. For all, bars represent average and error bars represent s.e.m. Source data

Our results suggest that WT H/NRas activity at the Golgi enables rebound MAPK signaling following Ras-G12C–GDP inhibition. To investigate the mechanisms for this Ras activation, we used Ras-LOCKR-PL (see Main for details) to measure the signaling environment of active Ras, which we term as the ‘signalosome’ (Fig. 2a). To profile the Ras signalosome at the Golgi, we localized the key of Ras-LOCKR-PL to the Golgi (Golgi Ras-LOCKR-PL) (Fig. 2a and Extended Data Fig. 5a). H358 cells expressing Golgi Ras-LOCKR-PL were cotreated with biotin and either AMG-510 or DMSO for 4 or 24 h. Cells were then lysed and biotinylated proteins were enriched with streptavidin beads, followed by tryptic digestion and label-free quantification with mass spectrometry (MS). AMG-510 treatment for 24 h led to greater protein enrichment compared to DMSO treatment in H358 and MIA PaCa-2 cells (Extended Data Fig. 5b,c), consistent with Golgi Ras-LOCKR-S data demonstrating that Golgi Ras activity increases after AMG-510 treatment (Fig. 1d). We calculated differences in enrichment values (labeled ‘difference’ in Fig. 2b, Extended Data Fig. 5b,c and Supplementary Table 1) of labeled proteins between 4-h and 24-h AMG-510 treatment, as hits more enriched after 24-h treatment may contribute to the beginning of rebound MAPK signaling. The proteins (PAPOLG (poly(A) polymerase-γ, MVP and VDAC1 (voltage-dependent anion channel 1)) demonstrating the largest differences between 4 and 24 h by our difference metric (see Fig. 2b legend for details of selection criteria) were verified for their in-cell biotinylation by a proximity ligation assay (PLA). In this experiment, PLA probes the colocalization between biotinylated proteins and a candidate protein of interest (POI) as a measure of a POI’s biotinylation by Golgi Ras-LOCKR-PL, which is detected as intracellular fluorescent puncta. Of note, we do not expect PLA puncta to necessarily be Golgi localized as the biotin labeling times are hours long and proteins are freely diffusing. Our PLA data confirmed that AMG-510 treatment increased biotinylation of PAPOLG, VDAC1 and MVP in H358 cells (Fig. 2c and Extended Data Figs. 5d and 6a), corroborating the data from our MS profiling experiments (Fig. 2b). We also observed that 24-h AMG-510 treatment led to increased biotinylation of immunoprecipated MVP, further validating our PLA results (Extended Data Fig. 6b). Of these three Golgi Ras signalosome components, only MVP knockdown (KD) abrogated AMG-510-promoted Golgi Ras activity in H358 cells (Fig. 2d and Extended Data Fig. 5e,f), suggesting that MVP contributes to AMG-510-promoted Golgi Ras activation.

MVP is a ubiquitous protein with unknown function but previous studies demonstrated a role in intracellular signaling and increased expression during chemotherapy and radiotherapy^19^. We observed that enrichment of MVP in the Golgi Ras signalosome may be because of the increased presence of MVP at juxta-Golgi regions (Extended Data Fig. 5g; see Discussion for our working model). Within 24 h, MVP levels also increased in KRas-G12C cells subjected to AMG-510 (Fig. 2e), consistent with a previous report that also saw increases in MVP levels after AMG-510 treatment^6^. Functionally, MVP KD completely abrogated AMG-510-resistant cell growth and colony formation in KRas-G12C cells (Extended Data Fig. 5h,i). MVP has been shown to regulate several signaling branches related to Ras through interactions with Src homology domain 2 (SH2)-containing protein tyrosine phosphatase 2 (Shp2) and Erk, to nuclearly sequester negative regulators of phosphatidylinositol 3-OH kinase (PI3K)–protein kinase B (Akt) signaling and, thus, activate Akt^20^ and to promote receptor tyrosine kinase (RTK)-mediated MAPK signaling^21^. However, the role and mechanisms of MVP in terms of drug resistance have not been thoroughly studied. While our data suggest that MVP has a local role in Golgi Ras activity, we first explored MVP’s role in global signaling. MVP KD eliminated rebound pErk in response to AMG-510 treatment and led to overall decreased pAkt levels (Fig. 2e). Altogether, these data show that MVP is required for local (Golgi Ras) and global (Erk and Akt) adaptive signaling in response to Ras-G12C–GDP inhibition.

### MVP facilitates the signaling adaptive cell subpopulation

**Fig. 3:**
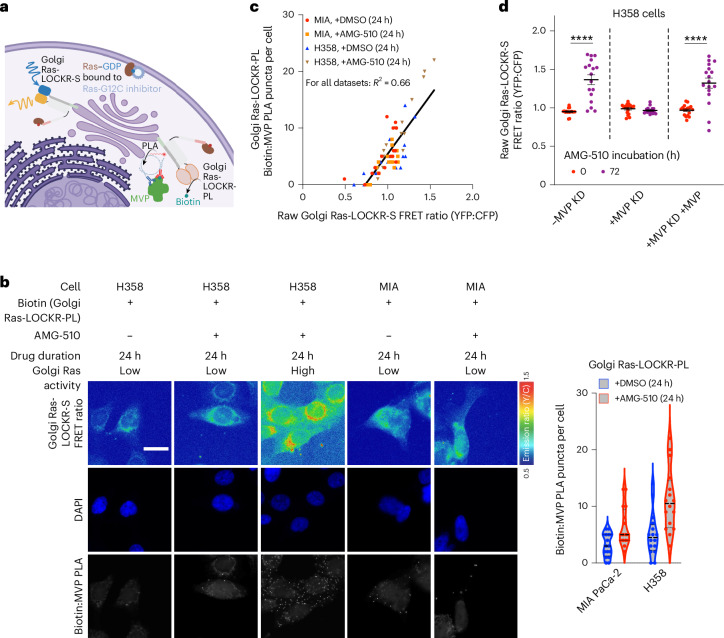
MVP at the Golgi facilitates the AMG-510-promoted signaling adaptive cell subpopulation. **a**, Schematic of the single-cell experiment relating Golgi Ras activity with the Golgi Ras signalosome. Golgi Ras-LOCKR-PL with PLA and Golgi Ras-LOCKR-S were used to relate MVP’s presence in the Golgi Ras signalosome to Golgi Ras activity, respectively, in response to AMG-510 treatment. **b**, Left, representative epifluorescence images from three biological replicates of H358 and MIA PaCa-2 cells expressing Golgi Ras-LOCKR-PL and Golgi Ras-LOCKR-S. Cells were cotreated with 500 μM biotin and either DMSO or 100 nM AMG-510 for 24 h. Cells were then fixed, subjected to PLA using anti-biotin and anti-MVP antibodies for PLA and then imaged for PLA puncta and FRET ratios. Right, violin plot comparing PLA puncta per cell (*n* = 16 cells). Each dot represents a single cell. The same dataset was used in Fig. 2c. CV for MIA PaCa-2 − AMG-510 = 66%, CV for MIA PaCa-2 + AMG-510 = 77%, CV for H358 − AMG-510 = 54% and CV for H358 + AMG-510 = 50%. Scale bar = 10 μm. **c**, Scatterplot comparing Golgi Ras-LOCKR-PL-mediated labeling of MVP (*y* axis) to Golgi Ras-LOCKR-S raw FRET ratios (*x* axis) in MIA PaCa-2 and H358 cells treated with DMSO or AMG-510 for 24 h. Each dot represents a single cell where both measurements were performed (*n* = 16 cells per condition). **d**, Raw FRET ratios of Golgi Ras-LOCKR-S in H358 cells transfected with MVP siRNA, scrambled siRNA or MVP siRNA followed by transfection with ectopic MVP 48 h later. Raw FRET ratios for 0 and 72 h treatment with 100 nM AMG-510 are shown (*n* = 17 cells per condition). Statistical analysis was conducted using a Student’s two-way *t*-test. *P* values, from left to right: 8.3 × 10^−6^ and 5.4 × 10^−6^. Source data

Given the effect of MVP on Golgi Ras activity, we wondered whether MVP’s presence in the Golgi Ras signalosome can explain the AMG-510-promoted signaling adaptive cell subpopulation. Thus, we used Golgi-localized Ras-LOCKR tools to link Golgi Ras activity to the Golgi Ras signalosome at the single-cell level. KRas-G12C cells expressing both Golgi Ras-LOCKR-S and Golgi Ras-LOCKR-PL were subjected to DMSO or AMG-510 for 24 h and underwent PLA analysis (Fig. 3a,b). Similar to pErk levels (Extended Data Fig. 1b), both Golgi Ras-LOCKR-PL-mediated biotinylation of MVP measured by PLA and Golgi Ras-LOCKR-S FRET ratios were heterogeneous in response to AMG-510 treatment (Fig. 3b and Extended Data Fig. 6a). This analysis demonstrated that the subpopulation of cells with high Golgi Ras activity (AMG-510-promoted signaling adaptive cell subpopulation) are typified by the presence of MVP in the Golgi Ras signalosome as Golgi Ras-LOCKR-S FRET ratios and PLA puncta representing MVP biotinylation by Golgi Ras-LOCKR-PL were positively correlated (*R*^2^ = 0.66) (Fig. 3c). This correlation did not hold when looking at MVP expression levels (Extended Data Fig. 6c). MVP KD suppressed Golgi Ras activity 72 h after AMG-510 treatment (Fig. 3d and Extended Data Fig. 6d–f), further confirming MVP’s functional role in facilitating AMG-510-promoted Golgi Ras activation. Thus, these results reveal MVP as an upstream effector of AMG-510-promoted Golgi Ras activation in the signaling adaptive cell subpopulation.

### MVP colocalizes with MAPK pathway components

**Fig. 4:**
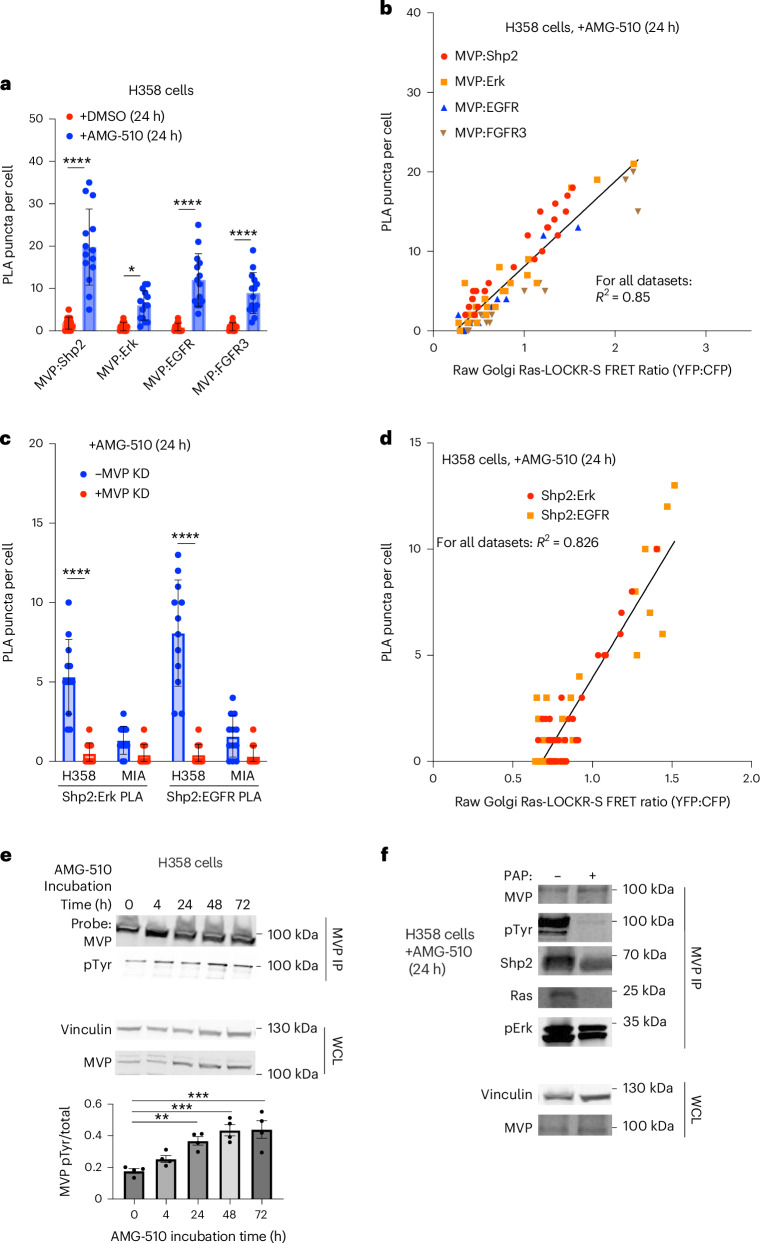
Single-cell signaling analysis shows that MVP colocalization with MAPK pathway components facilitates the AMG-510-promoted signaling adaptive cell subpopulation. **a**,**b**, H358 or MIA PaCa-2 cells were transfected with Golgi Ras-LOCKR-S and treated with DMSO or 100 nM AMG-510 for 24 h. Cells were then fixed and probed for MVP–MAPK pathway component proximity with PLA. Quantifications of different MVP–MAPK colocalizations (number of puncta per cell) (**a**) and the correlation between Golgi Ras-LOCKR-S raw FRET ratios and PLA puncta (anti-MVP and anti-MAPK pathway component) in cells (**b**) (*n* = 14 cells per condition). The same datasets were used in **a**,**b**. Statistical analysis was conducted using a Student’s two-way *t*-test. *P values, from left to right: 5.1 × 10^−6^, 0.032, 7.1 × 10^−6^ and 1.3 × 10^−6^. **c**,**d**, H358 or MIA PaCa-2 cells were transfected with MVP or scrambled siRNA, 2 days later transfected with Golgi Ras-LOCKR-S,and then incubated with 100 nM AMG-510 for 24 h. Cells were then fixed and underwent PLA analysis to probe for Shp2–Erk and Shp2–EGFR colocalization. Quantifications of Shp2–Erk and Shp2–EGFR interactions (number of punta per cell) (**c**) and correlation between Golgi Ras-LOCKR-S FRET ratios and PLA puncta (anti-Shp2, anti-Erk and anti-EGFR) in cells (**d**) (*n* = 12 cells per condition). The same datasets were used in **c**,**d**. Statistical analysis was conducted using a Student’s two-way *t*-test. *P* values, from left to right: 2.5 × 10^−6^ and 5.2 × 10^−7^. **e**, H358 cells were treated with 100 nM AMG-510 for 72 h followed by lysis, MVP immunoprecipitation and immunoblotting for total MVP and phosphorylated tyrosine levels. Top, representative immunoblots of immunoprecipitated (top) and whole-cell lysate samples (bottom). Bottom, densitometry quantification of immunoblots showing the ratio of MVP with the phosphorylated tyrosine modification (pTyr:MVP) (*n* = 4 biological replicates). The bar represents the average of four replicates and error bars represent the s.e.m. Statistical analysis was conducted using a Student’s two-way *t*-test. *P values, from left to right: 3.2 × 10^−3^, 8.7 × 10^−4^ and 4.4 × 10^−4^. **f**, H358 cells were treated with 100 nM AMG-510 for 24 h followed by lysis, incubation with or without PAP for 45 min, immunoprecipitation of MVP and immunoblotting for levels of coimmunoprecipitated Shp2, Ras and pErk. Representative immunoblots are shown with immunoprecipitation samples (top) and whole-cell lysate samples (bottom) (*n* = 3 experiments). Source data

**Extended Data Fig. 7:**
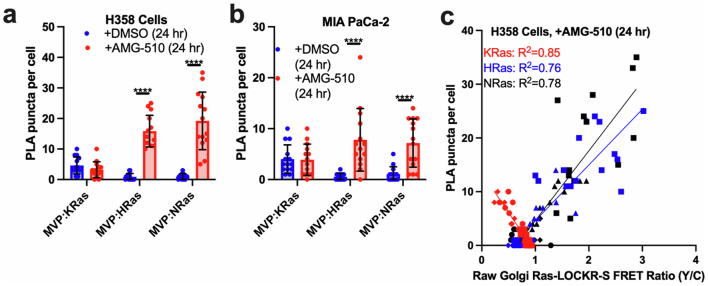
MVP co-localizations with Ras isoforms change during AMG-510 inhibition. (**a-c**) H358 (**a**) or MIA PaCa-2 (**b**) cells transfected with golgi-Ras-LOCKR-S and then treated with either DMSO or 100 nM AMG-510 for 24 hours were probed for MVP:Ras isoform co-localization via PLA. PLA puncta per cell for MVP:Ras isoforms in H358 (**a**) and MIA PaCa-2 (**b**) cells. Bars represent average from three biological replicates and error bars represent s.e.m. and (**c**) correlation between golgi-Ras-LOCKR-S raw FRET ratios and PLA puncta (anti-MVP, anti-KRas, anti-HRas, anti-NRas) per cell (n = 14 cells per condition, n = 42 cells in total per cell line). The same dataset in Extended Data Fig. 7c is shown in Extended Data Fig. 7a, b. Statistics: Student’s two-way t-test. *p-values, from left to right: 4.5 × 10^−5^, 5 × 10^−7^, 8.3 × 10^−6^, 5.6 × 10^−6^. Source data

We next investigated the molecular details underlying how MVP can activate Ras and its downstream MAPK pathway during AMG-510 treatment. MVP binds to Shp2 and Erk upon EGFR stimulation^21^. Given this link, we investigated MVP’s proximity with MAPK pathway components (Shp2, Erk and the RTKs EGFR and fibroblast growth factor receptor (FGFR)) in relation to Golgi Ras activities at the single-cell level. Using PLA this time to measure protein–protein proximities, H358 cells treated with AMG-510 for 24 h displayed increases in MVP’s colocalization with Shp2, Erk, EGFR and FGFR3 (Fig. 4a). Our single-cell interactome signaling analysis showed that MVP’s colocalization with Shp2, Erk, EGFR and FGFR3 positively correlated with Golgi Ras activity (*R*^2^ = 0.85) (Fig. 4b). Moreover, MVP KD in KRas-G12C cells decreased AMG-510-promoted Shp2–Erk and Shp2–EGFR colocalizations (Fig. 4c), suggesting that MVP facilitates scaffolding between Shp2 and its substrates. At the single-cell level, Shp2’s colocalization with Erk and EGFR was positively correlated (*R*^2^ = 0.83) with Golgi Ras activities (Fig. 4d)^22^. Given the functional role of MVP in Golgi Ras activity (Fig. 3d), we wondered whether MVP is in proximity with Ras itself. Using the best available antibodies for the different Ras isoforms^23^, we observed that MVP’s colocalization with WT H/NRas increased upon AMG-510 treatment but this was not the case for KRas (Extended Data Fig. 7a,b). At the single-cell level, MVP’s colocalization with H/NRas correlated well with Golgi Ras activity but KRas–MVP colocalization was negatively correlated with Golgi Ras activity (Extended Data Fig. 7c) presumably because of H/NRas but not KRas being endomembrane localized. These results suggest that MVP colocalizes with multiple MAPK signaling components to promote their adaptive signaling in response to Ras-G12C–GDP inhibition.

We next investigated how MVP associates with MAPK signaling components. EGFR stimulation leads to MVP tyrosine phosphorylation, leading to Shp2 recruitment through its SH2 domain^21^; thus, we probed whether MVP becomes tyrosine phosphorylated in response to AMG-510 treatment^17^. Indeed, we found that AMG-510 treatment led to a steady increase in MVP tyrosine phosphorylation (Fig. 4e). To test whether tyrosine phosphorylation is necessary for MVP’s interaction with MAPK pathway components, H358 cells were treated with AMG-510 for 24 h and the lysate was subjected to potato acid phosphatase (PAP) treatment^24^ followed by MVP immunoprecipitation and immunoblotting. PAP treatment led to decreased Shp2, Ras and pErk coimmunoprecipitation with MVP (Fig. 4f), suggesting that MVP’s interaction with MAPK components is mediated through phosphorylation.

Altogether, by pairing localized Ras signalosome profiling (Ras-LOCKR-PL) with subcellular Ras activities (Ras-LOCKR-S), we revealed how the AMG-510-promoted signaling adaptive cell subpopulation can arise. Our data suggest that, by bringing substrates and clients of the same pathway together, MVP-dependent scaffolding of MAPK components (Shp2, Erk, RTKs and WT H/NRas) enhances Golgi Ras activity, which promotes adaptive oncogenic signaling.

### AMG-510 alters metabolism and mitochondrial Ras (mito-Ras) activity

**Fig. 5:**
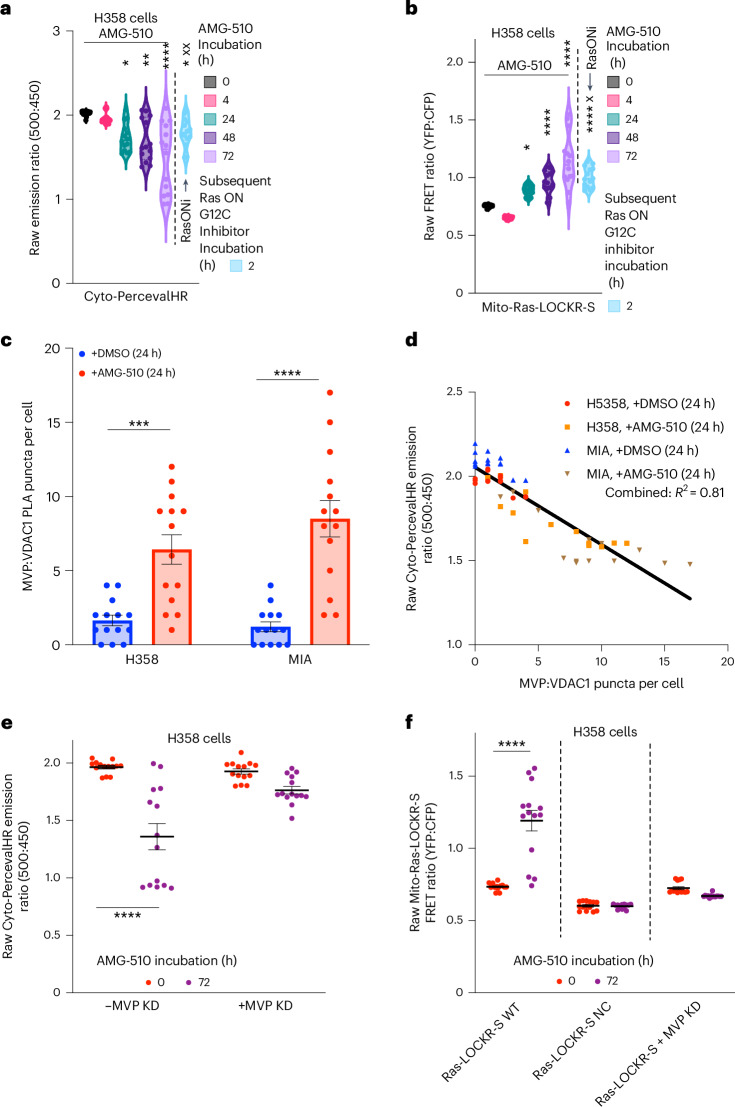
MVP facilitates the AMG-510-promoted metabolically adapting cell subpopulation. **a**,**b**, H358 cells transfected with either cyto-PercevalHR (**a**) or mito-Ras-LOCKR-S (localization sequence, N terminus of dual-specificity protein kinase A-anchoring protein 1) (**b**) were first treated with 100 nM AMG-510 alone for the times indicated or for 72 h with 100 nM AMG-510 followed by incubation with 100 nM RasONi for 2 h (*n* = 14 cells per condition). Shown are either the raw emission ratios (500 versus 450 nm) from cyto-PercevalHR (**a**) or raw FRET ratios from mito-Ras-LOCKR-S (**b**). Cyto-PercevalHR, CV at 0 h = 1.5%, CV at 4 h = 3.4%, CV at 24 h = 9.6%, CV at 48 h = 15%, CV at 72 h = 28% and CV after RasONi = 9.4%; mito-Ras-LOCKR-S, CV at 0 h = 1.6%, CV at 4 h = 2%, CV at 24 h = 5.2%, CV at 48 h = 9.8%, CV at 72 h = 20% and CV after RasONi = 8.8%. Statistical analysis was conducted using an ordinary two-way ANOVA. *P value comparison to 0-h AMG-510 time point, from left to right: 0.03, 2.3 × 10^−3^, 5.7 × 10^−6^ and 0.023 (**a**); 0.031, 9.7 × 10^−5^, 5 × 10^−8^ and 6.9 × 10^−7^ (**b**). ^x^*P* value comparison to 72-h AMG-510 time point, from left to right: 3.6 × 10^−3^ (**a**); 0.016 (**b**). **c**,**d**, H358 or MIA PaCa-2 cells were transfected with cyto-PercevalHR, incubated with DMSO or 100 nM AMG-510 for 24 h, fixed, underwent PLA analysis (anti-MVP, anti-VDAC1) and then imaged. Quantifications of MVP–VDAC1 interactions (number of puncta per cell) (**c**) and the correlation between cyto-PercevalHR emission ratios and PLA puncta (anti-MVP and anti-VDAC1) per cell (**d**) (*n* = 14 cells per condition). The same datasets were used in **c**,**d**. Statistical analysis was conducted using a Student’s two-way *t*-test. *P values, from left to right: 3.2 × 10^−4^ and 6.7 × 10^−6^. **e**, Raw emission ratios of H358 cells transfected with scrambled siRNA or MVP siRNA and then cyto-PercevalHR, followed by treatment for 72 h with 100 nM AMG-510 (*n* = 14 cells per condition). Statistical analysis was conducted using a Student’s two-way *t*-test. *P = 9.1 × 10^−7^. **f**, Raw FRET ratios of H358 cells transfected with mito-Ras-LOCKR-S WT, mito-Ras-LOCKR-S negative control biosensor or MVP siRNA and then mito-Ras-LOCKR-S WT before treatment for 72 h with 100 nM AMG-510 (*n* = 14 cells per condition). Statistical analysis was conducted using a Student’s two-way *t*-test. *P = 3.2 × 10^−6^. Source data

**Extended Data Fig. 8:**
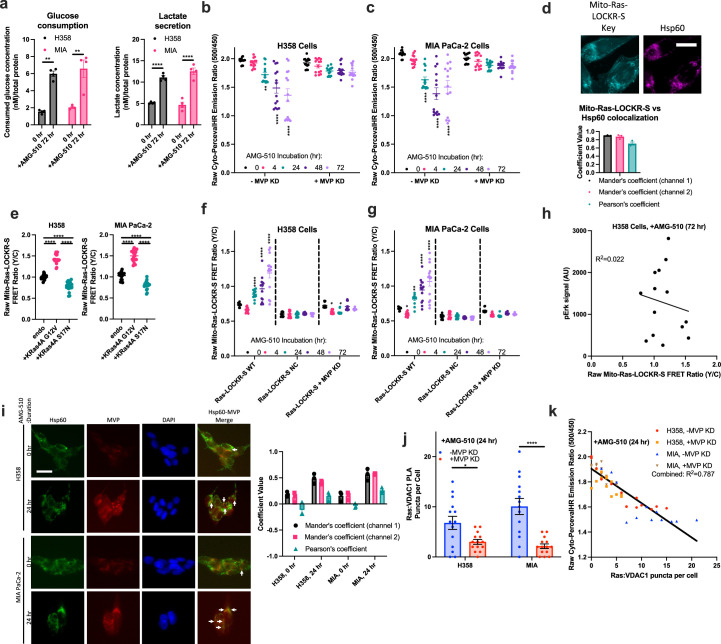
MVP is necessary for metabolic changes during AMG-510 treatment. (**a**) Glucose consumption and lactate release in untreated H358 and MIA PaCa-2 cells or treated with 100 nM AMG-510 for 24 h (n = 4 experiments). Statistics: student’s two-way t-test. *p-values, left to right: 6.4 × 10^−3^, 6.2 × 10^−3^, 5.7 × 10^−5^,6.6 × 10^−6^. (**b,c**) Raw emission ratios of H358 (**b**) or MIA PaCa-2 (**c**) cells transfected with either scrambled or MVP siRNA and then cyto-PercevalHR, followed by treating with 100 nM AMG-510 for the time indicated (n = 14 cells per condition). Statistics: Ordinary two-way ANOVA. *p-values are comparison to 0-hour time point, left to right: (**b**) 5.1 × 10^−3^, 6.3 × 10^−7^, 8 × 10^−7^, (**c**) 5.5 × 10^−5^, 4.8 × 10^−6^, 5.7 × 10^−7^. (**d**) Top: Representative image of H358 cells transfected with mito-Ras-LOCKR-S and immunostained for Hsp60. Bottom: Co-localization analysis between mito-Ras-LOCKR-S Key and Hsp60 (n = 3 experiments). Scale bar = 10 μm. (**e**) Raw FRET ratios of H358 and MIA PaCa-2 cells transfected with mito-Ras-LOCKR-S and either transfected with nothing else, KRas4A G12V or S17N^11^ (n = 20 cells per condition). Statistics: Ordinary two-way ANOVA. *p-values, left to right: 5.5 × 10^−6^, 8.1 × 10^−10^, 7.2 × 10^−6^, 8.5 × 10^−7^, 9.1 × 10^−11^, 3.2 × 10^−6^. (**f**,**g**) Raw FRET ratios of H358 (**f**) or MIA PaCa-2 (**g**) transfected with either mito-Ras-LOCKR-S WT, mito-Ras-LOCKR-S NC, or MVP siRNA and then mito-Ras-LOCKR-S WT, followed by 100 nM AMG-510 treatment for the times indicated (n = 14 cells per condition). Statistics: Ordinary two-way ANOVA. *p-values are comparison to 0-hour time point, left to right: (**f**) 5.3 × 10^−5^, 6.1 × 10^−6^, 1.4 × 10^−6^, 0.044, 0.04 (**g**) 0.041, 3.3 × 10−3,^−5^1.3 × 10^−6^, 7.5 × 10^−7^. (**h**) Single-cell scatterplot comparing mito-Ras-LOCKR-S raw FRET ratio to pErk immunostaining fluorescence in H358 cells treated with 100 nM AMG-510, fixed, immunostained, and imaged. (n = 14 cells). (**i**) Left: Representative images of H358 or MIA PaCa-2 cells treated with or without 100 nM AMG-510 and immunostained for MVP and Hsp60. Right: Co-localization analysis between MVP and Hsp60 (n = 3 experiments). Scale bar = 10 μm. (**j**,**k**) H358 or MIA PaCa-2 cells transfected with scrambled siRNA or MVP siRNA and subsequently transfected with Cyto-PercevalHR, treated with 100 nM AMG-510, fixed underwent PLA analysis (anti-pan-Ras, anti-VDAC1), and then imaged. (**j**) Quantifications of MVP:VDAC1 PLA per cell. (**k**) Single-cell scatterplot comparing cyto-PercevalHR emission ratios and Ras:VDAC1 PLA puncta per cell (n = 14 cells per condition). The same datasets were used in **j**,**k**. Statistics: Student’s two-way t-test. *p-values, left to right: 0.015, 6 × 10^−6^. For all, bars represent average and error bars represent s.e.m. Shared datasets between: Fig. 5e/Extended Data Fig. 8b, Fig. 5f/Extended Data Fig. 8f. Source data

Interestingly, mitochondrial VDAC1 and VDAC2 are two of the most prominently labeled proteins by Golgi Ras-LOCKR-PL 24 h after AMG-510 treatment (Fig. 2b). VDACs are outer mitochondrial membrane channel proteins that regulate the cytosolic and mitochondrial levels of adenosine triphosphate (ATP), which affects glycolytic rates as cytosolic ATP inhibits several glycolytic enzymes^25^. Also affecting glycolysis, KRas4A enhances glycolytic flux by blocking allosteric inhibition of hexokinase^12^. Recent reports indicated that Ras-G12C–GDP inhibition leads to increased mRNA and protein levels of glycolytic enzymes, with a concomitant increase in several glycolytic metabolites^6,8^. Consistent with these findings, we observed that treating KRas-G12C cells with AMG-510 for 72 h led to increased glucose consumption and lactate release, signatures of enhanced glycolytic flux (Extended Data Fig. 8a). To determine whether VDAC function is altered during AMG-510 treatment as a possible mechanistic link to the observed glycolytic changes, we used the fluorescence-based biosensor Cyto-PercevalHR^26,27^, which measures cytosolic ratios of ATP to adenosine diphosphate (ADP) (fluorescence emission ratios at 500 and 450 nm, respectively) at the single-cell level. We observed a heterogenous (H358 cells, CV at 72 h = 28%) reduction in cytosolic ATP:ADP ratios (Fig. 5a and Extended Data Fig. 8b,c) during AMG-510 treatment, suggesting that Ras-G12C–GDP inhibition alters VDAC function. Thus, Ras-G12C–GDP inhibition leads to a ‘metabolically adaptive subpopulation’ akin to the signaling adaptive cell subpopulation.

We suspected that the AMG-510-promoted upregulation of glycolysis (Fig. 5a) is due to its modulation of the KRas4A spliceform. KRas4A is the only Ras isoform localized at the outer mitochondrial membrane^12^; thus, as a proxy for mito-Ras activities, we measured mito-Ras–GTP levels by localizing the key of Ras-LOCKR-S to the outer mitochondrial membrane (Extended Data Fig. 8d,e). AMG-510 treatment led to an initial decrease (4 h) followed by a gradual heterogenous increase (H358 cells, CV at 72 h = 20%) in mito-Ras-LOCKR-S FRET ratios that exceeded basal levels (Fig. 5b and Extended Data Fig. 8f,g), which we interpreted as an initial block of basal mutant KRas4A activity, but then a significant increase in KRas4A–GTP levels possibly because of adaptive overexpression of mutant KRas^5^. As a control, no FRET ratio increases were seen in a negative control mito-Ras-LOCKR-S biosensor^11^ (Extended Data Fig. 8f,g) that possessed a mutant RasBD incapable of binding Ras–GTP^11,28^. Unlike Golgi Ras activity, mito-Ras activity did not correlate with global pErk levels (Extended Data Fig. 8h), suggesting that mito-Ras activity is not responsible for the AMG-510-promoted signaling adaptive cell subpopulation. To investigate whether WT or mutant Ras is responsible for these changes in cytosolic ATP:ADP ratios and mito-Ras activities, RasONi was added 72 h after AMG-510 treatment in KRas-G12C cells. Subsequent RasONi treatment for 2 h led to decreased mito-Ras activity and increased cytosolic ATP:ADP ratios (^x^*P* value comparison in Fig. 5a,b), suggesting that mutant KRas4A–GTP contributes to the observed mito-Ras activation and metabolic alterations that result from Ras-G12C–GDP inhibition.

### MVP is required for AMG-510-promoted metabolic changes

After establishing an AMG-510-promoted metabolically adaptive cell subpopulation (Fig. 5a and Extended Data Fig. 8a), we determined what facilitates this subpopulation. As MVP promotes Golgi Ras activation during AMG-510 treatment by colocalizing with MAPK pathway components (Fig. 4), we wondered whether MVP also has a role in altering mito-Ras activities and cytosolic ATP:ADP ratios. Hence, we again performed single-cell interactome and metabolism analysis using PLA and cyto-PercevalHR, respectively. We observed that AMG-510 treatment resulted in increased mitochondrial localization of MVP and MVP–VDAC1 colocalization after AMG-510 addition (Fig. 5c and Extended Data Fig. 8i) and that these colocalizations were inversely correlated with cytosolic ATP:ADP ratios (*R*^2^ = 0.81) (Fig. 5d). MVP also appeared to be required for AMG-510-promoted Ras–VDAC1 colocalizations as MVP KD led to decreased Ras–VDAC1 colocalization and these colocalizations were inversely correlated (*R*^2^ = 0.79) with cytosolic ATP:ADP ratios (Extended Data Fig. 8j,k). We observed that MVP is also necessary for AMG-510-promoted changes in metabolism as no changes in cytosolic ATP:ADP ratios were observed upon AMG-510 treatment of MVP KD cells (Fig. 5e and Extended Data Fig. 8b,c). MVP also seems to be required for adaptive mito-Ras signaling as MVP KD blunted AMG-510-promoted mito-Ras activation (Fig. 5f and Extended Data Fig. 8f,g). Overall, our single-cell analyses revealed that MVP is necessary for AMG-510-promoted metabolical adaptions in a subpopulation of cells by mediating the regulation of KRas4A and VDAC (see Discussion for potential mechanisms).

### Ras-G12C–GTP inhibition leads to MVP-dependent Ras signaling

**Fig. 6:**
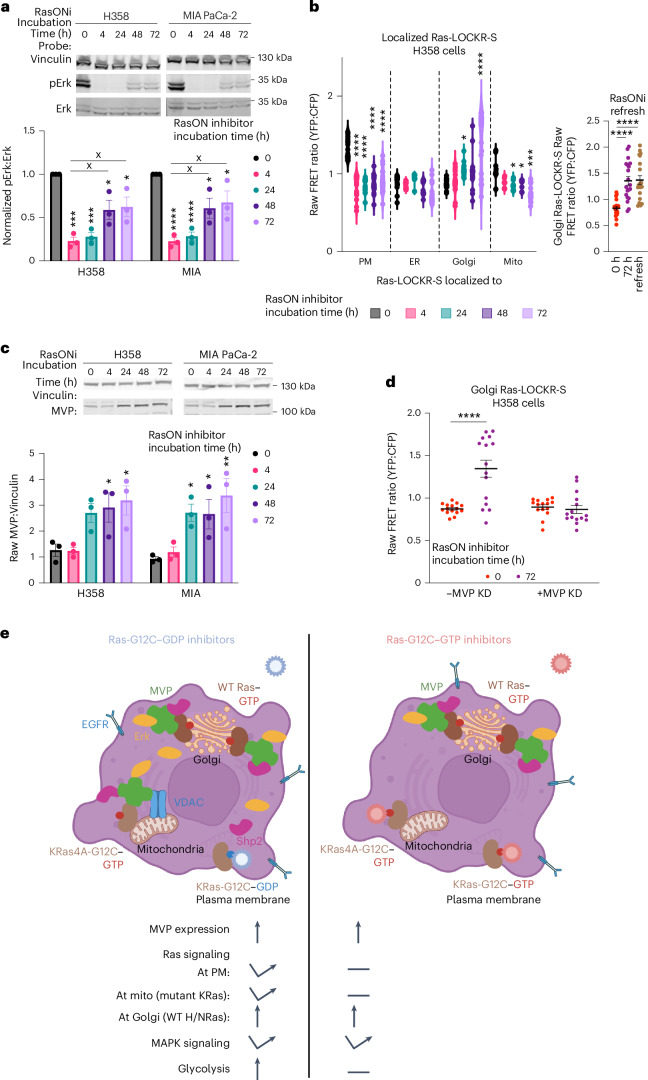
RasONi leads to MVP-mediated rebound oncogenic signaling. **a**, H358 and MIA PaCa-2 cells were treated with 100 nM RasONi for the indicated times and then underwent immunoblotting (*n* = 3 experiments). Top, representative immunoblot. Bottom, densitometry quantification of immunoblots where pErk:Erk ratios were calculated. Statistical analysis was conducted using an ordinary two-way ANOVA. *P value comparison to 0-h time point, from left to right: H358, 9.1 × 10^−4^, 3.2 × 10^−4^, 0.045 and 0.033; MIA PaCa-2, 7.6 × 10^−4^, 8.1 × 10^−4^, 0.023 and 0.045. ^x^*P* value comparison to 4-h RasONi time point, from left to right: H358, 0.037 and 0.016; MIA PaCa-2, 0.04 and 0.021. **b**, Left, raw FRET ratios of H358 cells transfected with subcellularly localized Ras-LOCKR-S and treated for indicated times with 100 nM RasONi (*n* = 15 cells per condition). Statistical analysis was conducted using an ordinary two-way ANOVA in comparison to 0-h time point. PM, CV at 0 h = 9.9%, 4 h = 19%, 24 h = 24%, 48 h = 27% and 72 h = 19%. ER, CV at 0 h = 11%, 4 h = 4.9%, 24 h = 7.5%, 48 h = 11% and 72 h = 19%. Golgi, CV at 0 h = 7.1%, 4 h = 17%, 24 h = 8.4%, 48 h = 24% and 72 h = 29%. Mitochondria, CV at 0 h = 15%, 4 h = 6.9%, 24 h = 7.3%, 48 h = 8.1% and 72 h = 14%. Right, raw FRET ratios of Golgi Ras-LOCKR-S-transfected H358 cells treated for 72 h with 100 nM RasONi before 2-h refresh (labeled ‘refresh’) of 100 nM RasONi. Statistical analysis was conducted using an ordinary two-way ANOVA. *P value comparison to 0-h time point or as otherwise indicated, from left to right: PM, 4.5 × 10^−7^, 5.1 × 10^−7^, 5.2 × 10^−6^ and 3.8 × 10^−6^; Golgi, 0.021 and 8.4 × 10^−5^; mitochondria, 0.015, 0.016 and 3.4 × 10^−4^; refresh, 6.6 × 10^−7^ and 7.7 × 10^−6^. **c**, H358 and MIA PaCa-2 cells were treated with 100 nM RasONi over the indicated times and then underwent immunoblotting (*n* = 3 experiments). Top, representative immunoblot. Bottom, densitometry quantification of immunoblots where MVP-to-Vinculin ratios were calculated. Statistical analysis was conducted using an ordinary two-way ANOVA. *P value comparison to 0-h time point, from left to right: H358, 0.035 and 0.047; MIA PaCa-2, 0.025, 0.04 and 8 × 10^−3^. **d**, Raw FRET ratios of H358 cells transfected with scrambled or MVP siRNA for 2 days before transfection with Golgi Ras-LOCKR-S and treatment for 72 h with 100 nM RasONi (*n* = 15 cells per condition). Statistical analysis was conducted using a Student’s two-way *t*-test. *P = 1.1 × 10^−5^. The -MVP KD dataset is the same as the Golgi-Ras-LOCKR-S dataset in **b**. **e**, Schematic of signaling mechanisms underlying Ras-G12C inhibitor resistance. Source data

**Extended Data Fig. 9:**
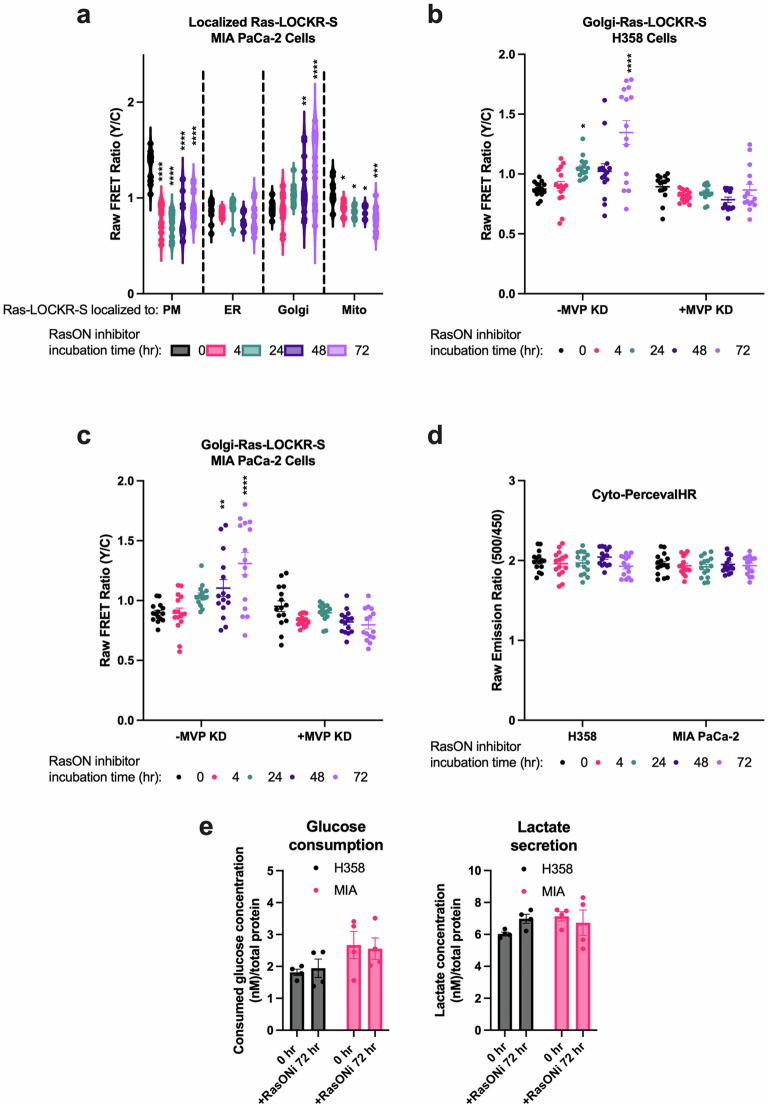
RasONi treatment induces compensatory oncogenic signaling that is dependent on MVP. (**a**) Raw FRET ratios of MIA PaCa-2 cells transfected with subcellularly localized Ras-LOCKR-S and treated over time with 100 nM RasONi (n = 15 cells per condition). Statistics: PM: CV at 0 hour = 12%, 4 hour = 19%, 24 hour = 18%, 48 hour = 28%, 72 hour = 18%. ER: CV at 0 hour = 12%, 4 hour = 5.2%, 24 hour = 8.6%, 48 hour = 10%, 72 hour = 19%. Golgi: CV at 0 hour = 8.6%, 4 hour = 18%, 24 hour = 8.8%, 48 hour = 26%, 72 hour = 28%. Mito: CV at 0 hour = 13%, 4 hour = 8.1%, 24 hour = 6.8%, 48 hour = 8.1%, 72 hour = 15%. Ordinary two-way ANOVA. *p-values are comparison to 0 hour time point, from left to right: PM: 7.2 × 10^−7^, 6.5 × 10^−6^, 4.3 × 10^−5^, 2.1 × 10^−5^, Golgi: 7.2 × 10^−3^, 6 × 10^−6^, Mito: 0.035, 0.031, 0.027, 3.4 × 10^−4^. (**b-c**) Raw FRET ratios of H358 (b) or MIA PaCa-2 (c) cells transfected either with scrambled or MVP siRNA for 2 days, then subsequently transfected with golgi-Ras-LOCKR-S, and treated over time with 100 nM RasONi (n = 15 cells per condition). Dataset in Extended Data Fig. 9b is the same as the one used in Fig. 6d. Statistics: Ordinary two-way ANOVA. *p-values are comparison to 0 hour time point, from left to right: (**b**) 0.042, 5.1 × 10^−5^, (**c**) 8.7 × 10^−3^, 2.9 × 10^−5^. (**d**) Raw emission ratios of H358 and MIA PaCa-2 cells transfected with cyto-PercevalHR and treated over time with 100 nM RasONi (n = 15 cells per condition). (**e**) H358 and MIA PaCa-2 cells were measured via commercial asays for glucose consumption and lactate release before or 72 hours after 100 nM RasONi addition (n = 4 experiments). Bars represent average from four biological replicates and error bars represent s.e.m. Source data

As RasONi^13^ targets the signaling competent form of Ras-G12C unlike AMG-510, we examined the downstream signaling that results from RasONi treatment. As Ras-G12C hydrolysis of GTP is slow, inhibitors of Ras-G12C–GTP should more quickly inhibit downstream signaling than Ras-G12C–GDP inhibitors, thus potentially limiting the timeframe for adaptive signaling to occur. Treatment of KRas-G12C cells with RasONi led to an initial decrease in pErk levels, but partial reactivation was observed after 48 and 72 h (Fig. 6a). Similar to AMG-510, RasONi treatment led to a significant and sustained reduction in detected Ras–GTP at the PM for up to 72 h and a substantial and heterogenous increase (CV at 72 h = 29%) in Golgi Ras activity, which was not altered when RasONi was refreshed (Fig. 6b and Extended Data Fig. 9a). Thus, RasONi appears to promote a signaling adaptive cell subpopulation similar to AMG-510. We previously saw that AMG-510 treatment led to upregulated MVP expression (Fig. 2e), which was required for AMG-510-promoted Golgi Ras activation (Fig. 3d); thus, we wondered whether RasONi leads to a similar effect. In KRas-G12C cells, MVP expression increased ~3-fold 72 h after RasONi treatment (Fig. 6c). MVP KD abrogated Golgi Ras activation in response to RasONi treatment (Fig. 6d and Extended Data Fig. 9b,c), suggesting that MVP has a similar role in RasONi-promoted signaling adaptations as it does for AMG-510.

In contrast to AMG-510, RasONi treatment led to prolonged decreases in mito-Ras-LOCKR-S FRET ratios (Fig. 6b and Extended Data Fig. 9a), demonstrating that RasONi shows sustained inhibition of mutant KRas4A activity at this subcellular location. Consistent with this result, no substantial changes in cytosolic ATP:ADP ratios at the single-cell level (Extended Data Fig. 9d) or bulk changes in glycolytic hallmarks (Extended Data Fig. 9e) were observed 72 h after RasONi treatment. Thus, KRas-G12C–GTP inhibition results in a clear difference in mitochondrial function relative to KRas-G12C–GDP inhibition, most likely because of the ability of RasONi to effectively inhibit mutant KRas4A at the mitochondria. Overall, there were similarities and differences between Ras-G12C–GDP inhibition (AMG-510) and Ras-G12C–GTP inhibition (RasONi) (Fig. 6e). Both inhibitors induced the expression of MVP to activate Golgi-localized WT Ras to compensate for mutant KRas inhibition. As a distinction between the inhibitors, mito-Ras activity and glycolysis were promoted only by AMG-510. In summary, application of our Ras-LOCKR tools captured a subpopulation of cells that reactivated oncogenic signaling (MAPK pathway) during both Ras-G12C–GDP and Ras-G12C–GTP inhibition, with MVP being necessary for this reactivation (Fig. 6e).

## Discussion

Unfortunately, many patients treated with Ras-G12C–GDP inhibitors relapse because of reactivation of the Ras–MAPK signaling pathway^4^. How cancer cells reactivate the same pathway that was initially inhibited is not completely clear. By analyzing Ras signaling changes at a single-cell level using our Ras-LOCKR tools, we detected KRas-G12C cell subpopulations with adaptive Ras signaling and rewired metabolism and identified their mechanisms. We demonstrate that WT H/NRas, especially at the Golgi, are key players in driving these oncogenic signaling adaptations, although WT KRas in heterozygous G12C lines (for example, H358) could play a role in Ras activation and rebound MAPK signaling. However, the contribution of WT KRas is likely not a deterministic factor, as previous studies showed that the levels of rebound MAPK signaling do not correlate with the presence or absence of WT KRas in KRas-G12C lines^7^. By relating signaling complex composition with Ras-LOCKR-PL and localized Ras activities with Ras-LOCKR-S at the single-cell level, we identified MVP as being necessary for the adaptations that a subpopulation of cancer cells adopt in response to Ras-G12C–GDP inhibition. However, our proximity labeler requires extended labeling times because of either the slow reconstitution and/or the low activity of split TurboID; thus, specific subcellular distribution information was likely lost. Furthermore, recently developed small molecules that inhibit Ras-G12C–GTP^13^ also led to signaling adaptations that are MVP dependent. A previous report^13^ demonstrated that refresh of RasONi in KRas-G12C cells led to decreased pErk levels; thus, our observed RasONi-promoted WT Golgi Ras activation may not lead to downstream Erk activation in this context. As several cancer therapies are associated with increased MVP expression, our results reaffirm the role of MVP in drug resistance and we wonder whether these MVP-mediated mechanisms may be generally involved in resistance to new inhibitors against other Ras mutants^29–31^ and perhaps other cancer drugs.

Mechanistically, at the Golgi, Ras-G12C–GDP inhibition promoted WT H/NRas signaling dependent on MVP colocalization with MAPK pathway proteins (Shp2, Erk, RTKs and Ras). Although we did not identify a Ras GEF in the Golgi Ras signalosome (Supplementary Table 1), MVP-mediated colocalization between Shp2 and Ras (Fig. 4) may increase Ras–GTP levels^32^. Alternatively, an MVP-dependent process could result in the accumulation of Ras–GTP at the Golgi, which can be sustained at the Golgi as there is less prevalent Ras GTPase activating protein activity at this subcellular location relative to the PM^33^. Another intriguing finding is that RTK signaling can affect Ras-G12C–GDP inhibitor-promoted Golgi Ras activation (Extended Data Fig. 3c), which may be due to EGFR’s communication with WT Ras during Ras-G12C inhibition and may involve MVP (Fig. 4); however, the mechanistic details are not fully flushed out. Another open question is whether these MVP-mediated effects are specific to endomembrane regions such as the mitochondria and Golgi. It is not clear whether MVP specifically accumulates at the Golgi (Extended Data Fig. 5g) or whether the increased expression of MVP (Fig. 2e) leads to overall accumulation of MVP at different regions, thus enhancing MVP binding and regulation of signaling molecules. Another mechanistic hole in our model is how Ras-G12C inhibition induces the increased MVP expression that is seen in our and other studies^6,8^ using in vitro KRas-G12C cell cultures. As MVP expression can also be stimulated by cytokines^34^, there is a need to investigate these phenomena in more physiologically relevant models.

At the mitochondria, Ras-G12C–GDP inhibition led to MVP-dependent decreases in cytosolic ATP:ADP ratios and KRas4A activation, resulting in increased glycolysis. We hypothesize that MVP-mediated scaffolding of signaling components and VDAC1 promotes KRas4A–GTP and facilitates cross-talk between Ras signaling and glycolysis^12^ (Fig. 5 and Extended Data Fig. 8j,k); however, further validation of this hypothesis is needed. At a molecular level, MVP can colocalize with KRas (Extended Data Fig. 7), mitochondrial KRas4A regulates glycolysis through hexokinase^12^ and VDAC is known to interact with hexokinase^25^. Thus, an MVP–KRas4A–hexokinase–VDAC multiprotein complex may exist. As MVP also interacts with MAPK pathway components such as Erk (Fig. 4a,b,f) and activated Erk can phosphorylate and regulate VDAC channel opening^35^, MVP-localized Erk regulating VDAC function may be one possible mechanism.

Using our new biosensors and biochemical assays, we performed single-cell signaling analyses to identify and characterize a subpopulation of KRas-G12C-addicted cancer cells that evade several Ras-G12C inhibitors. We identified MVP as a key mediator of this drug-resistant subpopulation through its colocalization with MAPK pathway components and metabolite channels. MVP-mediated adaptations promote Ras signaling at endomembranes (Golgi and mitochondria), which enables downstream signaling and oncogenic metabolism. These results also illustrate how the proper spatiotemporal regulation of central biomolecules such as Ras is necessary for cellular homeostasis. Overall, this study introduces the technique of single-cell signaling analysis to understand complex biological phenomena such as drug resistance, thus facilitating the discovery of more key factors involved in pathogenesis.

## Methods

### Reagents

All reagents are listed in Supplementary Table 2.

### Cell culture and transfection

MIA PaCa-2 and H358 cells were cultured in DMEM containing 1 g l^−1^ glucose and supplemented with 10% (v/v) FBS and 1% (v/v) penicillin–streptomycin. All cells were grown in a humidified incubator at 5% CO_2_ and 37 °C.

Before transfection, all cells were plated onto sterile poly(d-lysine) coated plates or dishes and grown to 50–70% confluence. siRNA transfection used Lipofectamine RNAiMAX for at least 2 days. The remaining transfections used Fugene HD and cells were grown for an additional 16–24 h before subsequent experiments. All cells underwent serum starvation for at least 16 h before any treatment, unless indicated.

### Generation of stable cell lines

Generating localized Ras-S17N in H358 and MIA PaCa-2 cells involved lentiviral transduction. Lentiviruses were made by transfection of pLenti backbone versions of Ras-S17N with the packaging vectors pMD2.G (gift from D. Trono, Addgene plasmid 12259) and psPAX2 (gift from D. Trono, Addgene plasmid 12260) into HEK293T cells. At 24 h after transfection, HEK293T cells were replenished with fresh medium. After another 2 days, supernatant was collected and sterile-filtered through a 0.45-μm filter. Ras-S17N lentiviral solutions were added to H358 and MIA PaCa-2 cells. A negative control plate was also grown that was infected with an empty pLenti backbone. After another 2 days, 100 ng ml^−1^ puromycin was added until 95% cell death in the negative control plate. Cells were recovered without puromycin until cells were 80% confluent. Afterward, recombined stable cell lines were maintained in 1 μg ml^−1^ puromycin^36^.

### Plasmid construction

All plasmids were constructed using the pcDNA 3.1 backbone (unless otherwise indicated) and were produced by GenScript. All Ras-LOCKR plasmids and their respective sequences can be obtained through Addgene 216496-216507 (https://www.addgene.org/browse/article/28244240/).

### Cell counting to measure cell proliferation

H358 and MIA PaCa-2 cells were seeded in six-well plates at 10,000 cells per well. After 1 day of plating, cells were treated with the indicated drugs. Cell numbers were quantified using a hemacytometer each day for 7 days.

### Colony formation assay

H358 and MIA PaCa-2 cells were seeded in 24-well plates at 100 cells per well. After 1 day of plating, cells were treated with the indicated drugs. After 1–2 weeks to allow cell growth, cells were washed once with PBS, fixed with 4% paraformaldehyde (PFA) in PBS for 10 min, stained with 2.5 mg ml^−1^ crystal violet stain dissolved in 20% methanol for 10 min and then washed six times with PBS. Images were captured using the ZOE Fluorescent Cell Imager (BioRad).

### Immunostaining

H358 and MIA PaCa-2 cells were seeded onto 24-well glass-bottom plates. After transfection and drug addition, cells were fixed with 4% PFA in 2× PHEM buffer (60 mM PIPES, 50 mM HEPES, 20 mM EGTA, 4 mM MgCl_2_ and 0.25 M sucrose; pH 7.3) for 10 min, permeabilized with 100% methanol for 10 min, washed with PBS three times, blocked in 1% BSA in PBS for 30 min, incubated with primary antibody overnight at 4 °C, washed with PBS three times, incubated with DAPI and secondary antibodies for 1 h at room temperature and covered with aluminum foil. Cells were then washed with PBS three times and mounted for epifluorescence imaging. All images were analyzed in ImageJ.

### PLA

H358 and MIA PaCa-2 cells were seeded onto 24-well glass-bottom plates. After transfection and drug addition, cells were fixed with 4% PFA in 2× PHEM buffer for 10 min, permeabilized with 100% methanol for 10 min, washed with PBS three times, blocked in 1% BSA in PBS for 30 min, incubated with primary antibody overnight at 4 °C, washed with wash buffer A two times, incubated with DAPI and secondary antibody with PLUS or MINUS DNA for 1 h at 37 °C, washed with wash buffer A two times, incubated in ligation buffer for 30 min at 37 °C, washed with wash buffer A two times, incubated in amplification buffer for 100 min at 37 °C and finally washed with wash buffer B three times. Cells were then mounted for epifluorescence imaging. All images were analyzed in ImageJ.

### Immunoblotting and immunoprecipitation

Cells expressing the indicated constructs and incubated with the indicated drugs were plated, transfected and labeled as described in the figure legends. Cells were then transferred to ice and washed two times with ice-cold Dulbecco’s PBS (DPBS). Cells were then detached from the well by the addition of 1× radioimmunoprecipitation assay (RIPA) lysis buffer (50 mM Tris pH 8, 150 mM NaCl, 0.1% SDS, 0.5% sodium deoxycholate, 1% Triton X-100, 1× protease inhibitor cocktail, 1 mM PMSF, 1 mM Na_3_VO_4_ and 1% NP-40) and either scraping of cells or rotation on a shaker for 30 min at 4 °C. Cells were then collected and vortexed for at least 5 s every 10 min for 20 min at 4 °C. Cells were then collected and clarified by centrifugation at 20,000 r.p.m. for 10 min at 4 °C. The supernatant was collected and underwent a Pierce BCA assay to quantify total protein amounts.

For immunoblotting, whole-cell lysate protein amounts were normalized across samples in the same gel, mixed with 4× loading buffer before loading, incubated at 95 °C for 5 min and then 4 °C for 5 min and separated on any kDa SDS–PAGE gels. Proteins separated on SDS–PAGE gels were transferred to nitrocellulose membranes using the TransBlot system (BioRad). The blots were then blocked in 5% milk (w/v) in TBST (Tris-buffered saline and 0.1% Tween 20) for 1 h at room temperature. Blots were washed with TBST three times and incubated with the indicated primary antibodies in 1% BSA (w/v) in TBST overnight at 4 °C. Blots were then washed with TBST three times and incubated with LICOR dye-conjugated secondary antibodies (LICOR 680/800 or streptavidin–LICOR 800) in 1% BSA (w/v) in TBST for 1 h at room temperature. The blots were washed with TBST three times and imaged on an Odyssey IR imager (LICOR). Quantitation of Western blots was performed using ImageJ on raw images.

For immunoprecipitation, agarose beads were either preloaded with streptavidin (high-capacity streptavidin beads) or loaded by three lysis buffer washes before the addition of 1 mg ml^−1^ of the indicated antibodies at 4 °C on an orbital shaker for 3 h. Beads were then washed two times in lysis buffer. Whole-cell lysate protein amounts were normalized across samples and protein samples were added to beads (at least 100 μg per sample) either at room temperature for 1 h for streptavidin beads or at 4 °C on an orbital shaker overnight. Beads were then washed two times in lysis buffer and one time in TBS and then mixed with 4× loading buffer sometimes containing 2 mM biotin and 20 mM DTT^37^ for streptavidin pulldowns. The remaining portion of the protocol was the same as for immunoblotting.

### MS analysis

Cells expressing the indicated constructs and incubated with the indicated drugs were plated, transfected and labeled as described in figure legends. Cells were then transferred to ice and washed two times with ice-cold DPBS, detached from the well by the addition of 1× RIPA lysis buffer (50 mM Tris pH 8, 150 mM NaCl, 0.1% SDS, 0.5% sodium deoxycholate, 1% Triton X-100, 1× protease inhibitor cocktail, 1 mM PMSF, 1 mM Na_3_VO_4_ and 1% NP-40) and scraping of cells, collected and vortexed for at least 5 s every 10 min for 20 min at 4 °C and collected and clarified by centrifugation at 20,000*g* for 10 min at 4 °C. The supernatant was collected and underwent a Pierce BCA assay to quantify total protein amounts.

Next, 50 μl of high-capacity streptavidin agarose beads were washed two times in lysis buffer. Whole-cell lysate protein amounts were normalized across samples and protein samples were added to beads (at least 100 μg per sample) at room temperature for 1 h. Beads were then washed two times with lysis buffer, one time with 1 M KCl, one time with 0.1 M Na_2_CO_3_, two times with 2 M urea and two times with TBS. Beads were resuspended in 50 μl of denaturing buffer (6 M guanidinium chloride, 50 mM Tris containing 5 mM TCEP and 10 mM CAM with TCEP and CAM added fresh every time), inverted a few times and heated to 95 °C for 5 min. The bead slurry was diluted with 50 μl of 100 mM TEAB and 0.8 μg of LysC was added per sample with the pH adjusted to 8–9 using 1 M NaOH. This mixture was agitated on a thermomixer at 37 °C for 2 h at 1,400 r.p.m. Afterward, samples were diluted two times with 100 μl of 100 mM TEAB with 0.8 μg of sequencing-grade trypsin per sample, with the pH adjusted to 8–9 using 1 M NaOH. This mixture was agitated on a thermomixer at 37 °C for 12–14 h at 800 r.p.m. After overnight trypsinization, samples were diluted two times with 200 μl of buffer A (5% acetonitrile with 0.1% TFA) containing 1% formic acid. These samples were inverted a few times, with the pH adjusted to 2–3 using 100% formic acid. StageTips for peptide desalting were prepared by extracting plugs from C18 matrices, shoving them down a 200-μl tip and pressing them with a plunger for flatness. Using these StageTips, 50 μl of buffer B (80% acetonitrile with 0.1% TFA) was passed through at 4,000*g* for 1 min followed by 50 μl of buffer A at 4,000*g* for 1 min. The supernatant of the samples was added to StageTips and spun down at 4,000*g* for 5 min. Then, 50 μl of buffer A was added and spun down at 4,000*g* for 2.5 min. Next, 50 μl of buffer B was added to stage tips and a syringe pump was applied to elute samples.

Peptide samples were separated on an EASY-nLC 1200 System (Thermo Fisher Scientific) using 20-cm-long fused silica capillary columns (100 µm inner diameter, laser pulled in-house with Sutter P-2000, Novato) packed with 3-μm 120-Å reversed-phase C18 beads (Dr. Maisch). The liquid chromatography (LC) gradient was 90 min long with 5–35% B at 300 nl min^−1^. LC solvent A was 0.1% (v/v) aqueous acetic acid and LC solvent B was 20% 0.1% (v/v) acetic acid with 80% acetonitrile. MS data were collected with a Thermo Fisher Scientific Orbitrap Fusion Lumos using a data-dependent data acquisition method with an Orbitrap MS1 survey scan (R = 60 K) and as many Orbitrap HCD MS2 scans (R = 30 K) possible within the 2-s cycle time.

### Computation of MS raw files

Raw data files were analyzed by MaxQuant/Andromeda version 1.5.2.8 using protein, peptide and site false discovery rates of 0.01, a score minimum of 40 for modified peptides and 0 for unmodified peptides and a delta score minimum of 17 for modified peptides and 0 for unmodified peptides. MS/MS spectra were searched against the UniProt human database (updated July 22, 2015). MaxQuant search parameters were as follows: variable modifications, oxidation (M) and phosphorylation (S/T/Y); fixed modification, carbamidomethyl (C); maximum number of missed cleavages, 2; enzyme, trypsin/P; maximum charge, 7. The MaxQuant ‘match between runs’ feature was enabled. The initial search tolerance for FTMS scans was 20 ppm and 0.5 Da for ion trap MS/MS scans.

### MaxQuant output data processing

MaxQuant output files were processed, statistically analyzed and clustered using the Perseus software package version 1.5.6.0. Human gene ontology (GO) terms (biological process, cellular component and molecular function) were loaded from the ‘mainAnnot.homo_sapiens.txt’ file downloaded on March 2, 2020. Expression columns (protein and intensities) were log_2_-transformed and normalized by subtracting the median log_2_ expression value from each expression value of the corresponding data column. Potential contaminants, reverse hits and proteins only identified by site (biotinylation) were removed. Reproducibility between LC–MS/MS experiments was analyzed by column correlation (Pearson’s *r*) and replicates with a variation of *r* > 0.25 compared to the mean *r* values of all replicates of the same experiment were considered outliers and excluded from the analyses. Data imputation was performed in Perseus using a modeled distribution of MS intensity values downshifted by 1.8 and having a width of 0.2. Ratios in Fig. 2b are only shown for proteins that demonstrated selective labeling within KRas-G12C cells for either the 4-h or 24-h AMG-510 treatment datasets (more than twofold change (log_2_(ratio) > 1)) and passing a statistical cutoff (*P* value cutoff of 0.5 (−log_10_(*P* value) ≈ 1.3) for Student’s two-way *t*-test). Ubiquitous proteins were excluded. Hits were further filtered using GO analysis (signaling pathways) through the PANTHER database^38,39^.

### Glucose consumption and lactate release assays

H358 and MIA PaCa-2 cells were seeded on 24-well plates at 10,000 cells per well. Supernatant fractions were collected after 1 day of cell settling and 72 h after AMG-510 addition. Metabolite concentrations were quantified using the Lactate-Glo Assay and Glucose-Glo Assay according to the manufacturer’s instructions. Glucose consumption and lactate production were normalized to the total protein content, which was measured by the BCA protein assay.

### Time-lapse epifluorescence imaging

Drugs were added for the indicated durations. Cells were washed twice with FluoroBrite DMEM imaging medium and subsequently imaged in the same medium in the dark at room temperature. Epifluorescence imaging was performed on a Yokogawa CSU-X1 microscope with either a Lumencor Celesta light engine with seven laser lines (408, 445, 473, 518, 545, 635 and 750 nm) or a Nikon LUN-F XL laser launch with four solid-state lasers (405, 488, 561 and 640 nm), a ×40 (0.95 numerical aperture) objective and a Hamamatsu ORCA-Fusion scientific CMOS camera, controlled by NIS Elements 5.30 software (Nikon). The following excitation and emission filter combinations (center/bandwidth in nm) were used: CFP, 445 and 483/32; CFP–YFP FRET, 445 and 542/27; YFP, 473 and 544/24; GFP, 473 and 525/36; RFP, 545 and 605/52; far red (for example, AlexaFluor 647, 635 and 705/72; AlexaFluor 450, 445 and 525/36; Alexa Fluor 500, 488 and 525/36). Exposure times were 100 ms for the acceptor direct channel and 500 ms for all other channels, with no electronic multiplication gain set and no neutral-density filter added. Cells that were too bright (acceptor channel intensity 3 s.d. above the mean background intensity across experiments). All Ras-LOCKR-S plasmids contained a P2A sequence between the portions encoding the key and cage to help with roughly similar expression levels inside cells. There was no significant dependence of Ras-LOCKR-S expression on dynamic range^11^; thus, no fluorescence gating was performed. All epifluorescence experiments were subsequently analyzed using Image J. Bright-field images were acquired on the ZOE Fluorescent Cell Imager (BioRad).

For imaging multiday drug incubations, cells were first transfected with Ras-LOCKR-S or cyto-PercevalHR. Then, 1 day later, drugs were added with the 72-h time point being added first. During drug incubations, cells were housed in a humidified incubator at 5% CO_2_ and 37 °C. Each subsequent day, drugs were added as appropriate. After all the drug time points were added and the appropriate time elapsed, living cells were imaged under epifluorescence for around 10 min. Ras-LOCKR-S and cyto-PercevalHR time-course imaging were performed in living cells. For assays that required imaging/staining such as immunostaining or FRET imaging and PLA, cells were fixed and underwent the respective protocols.

### FRET biosensor analysis

Raw fluorescence images were corrected by subtracting the background fluorescence intensity of a cell-free region from the emission intensities of biosensor-expressing cells. CFP–YFP FRET ratios were then calculated at each time point (*R*). Graphs were plotted using GraphPad Prism 8 (GraphPad).

### Colocalization analysis

For colocalization analysis, cell images were individually thresholded and underwent Coloc 2 analysis on ImageJ. Mander’s coefficient is a measure of the fraction of intensity in one channel in pixels where there is above-threshold intensity in the other channel. Mander’s coefficient can only range from 0 to 1 where 0 represents no colocalization and 1 represents complete colocalization. Pearson’s coefficient is a ratio between the intensity of a pixel with background subtraction in both compared channels and can range from −1 to 1 where −1 represents a one-to-one negative correlation in the two channel’s pixel intensities and 1 represents one-to-one positive correlation in the two channel’s pixel intensities. A replicate in colocalization analysis is a whole-field epifluorescence image of cells.

### Quantification of PLA puncta

For the analysis of puncta number, cell images were individually thresholded and underwent particle analysis with circularity and size cutoffs in ImageJ.

### Statistics and reproducibility

No statistical methods were used to predetermine sample sizes but our sample sizes are similar to those reported in previous publications^11^. No sample was excluded from data analysis and no blinding was used. All data were assessed for normality. Data distribution was assumed to be normal but this was not formally tested. For normally distributed data, pairwise comparisons were performed using unpaired Student’s two-way *t*-tests, with Welch’s correction for unequal variances used as indicated. Comparisons among three or more groups were performed using ordinary one-way or two-way analyses of variance (ANOVAs) as indicated. All data shown are reported as the mean and error bars in the figures represent the s.e.m. All data were obtained at least in biological triplicates unless otherwise stated. All data were analyzed and plotted using GraphPad Prism 8 including nonlinear regression fitting. CV values were calculated as the s.d. divided by the mean.

### Reporting summary

Further information on research design is available in the Nature Portfolio Reporting Summary linked to this article.

## Online content

Any methods, additional references, Nature Portfolio reporting summaries, source data, extended data, supplementary information, acknowledgements, peer review information; details of author contributions and competing interests; and statements of data and code availability are available at 10.1038/s41589-024-01684-4.

## Supplementary information

Reporting Summary

Supplementary Tables 1 and 2 **Supplementary Table 1**: Differential Golgi Ras-LOCKR-PL labeling during AMG-510 treatment. H358 cells transfected with Golgi Ras-LOCKR-PL were incubated with 500 µM biotin and with or without 100 nM AMG-510 for 4 or 24 h. Afterwards, cells were lysed, underwent streptavidin pulldown, trypsin-digested and sent for MS analysis. Hits identified by MS were filtered on the basis of selectively labeling (more than twofold change compared to no AMG-510), passed our statistical cutoff and were related to signaling on the basis of GO analysis (see Methods for details). Statistical analysis was conducted using a Student’s two-way *t*-test. **Supplementary Table 2**: Reagents used throughout this study, including antibody dilutions.

## Source data

Source Data Fig. 1 Source data.

Source Data Fig. 2 Source data and unprocessed western blots.

Source Data Fig. 3 Source data.

Source Data Fig. 4 Source data and unprocessed western blots.

Source Data Fig. 5 Source data.

Source Data Fig. 6 Source data and unprocessed western blots.

Source Data Extended Data Fig. 1 Source data and unprocessed western blots.

Source Data Extended Data Fig. 2 Source data.

Source Data Extended Data Fig. 3 Source data and unprocessed western blots.

Source Data Extended Data Fig. 4 Source data and unprocessed western blots.

Source Data Extended Data Fig. 5 Source data and unprocessed western blots.

Source Data Extended Data Fig. 6 Source data and unprocessed western blots.

Source Data Extended Data Fig. 7 Source data.

Source Data Extended Data Fig. 8 Source data.

Source Data Extended Data Fig. 9 Source data.

## Data Availability

The data that support the findings of this study are available from figshare (10.6084/m9.figshare.25836802.v1)^40^. Proteomic raw data are available from PRIDE (https://massive.ucsd.edu/ProteoSAFe/dataset.jsp?accession=MSV000093478). Source data are provided with this paper.
