# Supplementary material for: Single-cell sensor analyses reveal signaling programs enabling Ras-G12C drug resistance
Source: Nat Chem Biol. 2024 Aug 5;21(1):47–58. doi: 10.1038/s41589-024-01684-4 (PMC11666463; doi:10.1038/s41589-024-01684-4)
Supplement: Supplementary file 1 — Reporting Summary [file 41589_2024_1684_MOESM1_ESM.pdf]

Corresponding author(s): Jason Z Zhang, Dustin J Maly

Last updated by author(s): May 17, 2024

Reporting Summary

Nature Portfolio wishes to improve the reproducibility of the work that we publish. This form provides structure for consistency and transparency in reporting. For further information on Nature Portfolio policies, see our Editorial Policies and the Editorial Policy Checklist.

Statistics

For all statistical analyses, confirm that the following items are present in the figure legend, table legend, main text, or Methods section.

n/a	Confirmed	
☐	☒	The exact sample size (*n*) for each experimental group/condition, given as a discrete number and unit of measurement
☐	☒	A statement on whether measurements were taken from distinct samples or whether the same sample was measured repeatedly
☐	☒	The statistical test(s) used AND whether they are one- or two-sided<br>*Only common tests should be described solely by name; describe more complex techniques in the Methods section.*
☒	☐	A description of all covariates tested
☒	☐	A description of any assumptions or corrections, such as tests of normality and adjustment for multiple comparisons
☐	☒	A full description of the statistical parameters including central tendency (e.g. means) or other basic estimates (e.g. regression coefficient) AND variation (e.g. standard deviation) or associated estimates of uncertainty (e.g. confidence intervals)
☐	☒	For null hypothesis testing, the test statistic (e.g. *F*, *t*, *r*) with confidence intervals, effect sizes, degrees of freedom and *P* value noted<br>*Give P values as exact values whenever suitable.*
☒	☐	For Bayesian analysis, information on the choice of priors and Markov chain Monte Carlo settings
☒	☐	For hierarchical and complex designs, identification of the appropriate level for tests and full reporting of outcomes
☐	☒	Estimates of effect sizes (e.g. Cohen's *d*, Pearson's *r*), indicating how they were calculated

Our web collection on statistics for biologists contains articles on many of the points above.

Software and code

Policy information about availability of computer code

Data collection	Epiluorescence images were captured through NIS Elements 5.30 software.
Data analysis	For analyzing raw mass spectrometry data, MaxQuant/Andromeda version 1.5.2.8 was used. Further statistical analysis of mass spectrometry data was processed using Perseus software package v1.5.6.0. Fiji 2.13.1 was used to analyze epifluorescence images. Data was analyzed and graphed on GraphPad Prism 8.

For manuscripts utilizing custom algorithms or software that are central to the research but not yet described in published literature, software must be made available to editors and reviewers. We strongly encourage code deposition in a community repository (e.g. GitHub). See the Nature Portfolio guidelines for submitting code & software for further information.

Data

Policy information about availability of data

All manuscripts must include a data availability statement. This statement should provide the following information, where applicable:

- Accession codes, unique identifiers, or web links for publicly available datasets
- A description of any restrictions on data availability
- For clinical datasets or third party data, please ensure that the statement adheres to our policy

Panther database was further used to cluster mass spectrometry hits. The data that support the findings of this study are available from Figshare (https://

figshare.com/articles/dataset/Combined_Data_for_Zhang_JZ_et_al_Nature_Chemical_Biology/25836802). Proteomic raw data is available on PRIDE (https://massive.ucsd.edu/ProteoSAFe/dataset.jsp?accession=MSV000093478).

Human research participants

Policy information about studies involving human research participants and Sex and Gender in Research.

Reporting on sex and gender	N/A
Population characteristics	N/A
Recruitment	N/A
Ethics oversight	N/A

Note that full information on the approval of the study protocol must also be provided in the manuscript.

Field-specific reporting

Please select the one below that is the best fit for your research. If you are not sure, read the appropriate sections before making your selection.

☒ Life sciences ☐ Behavioural & social sciences ☐ Ecological, evolutionary & environmental sciences

For a reference copy of the document with all sections, see nature.com/documents/nr-reporting-summary-flat.pdf

Life sciences study design

All studies must disclose on these points even when the disclosure is negative.

Sample size	All experiments were done in at least triplicate. The sample size is chosen based on sufficiency for statistical analysis for calculating assay specificity and sensitivity.
Data exclusions	No sample was excluded from data analysis.
Replication	The results were successfully replicated using different cell line stocks and on different days.
Randomization	Beyond expression of transfected plasmids, sample allocation for experimental groups was random. No further randomization was performed as this is not a treatment/response study. Regardless of covariation, the conclusions are the same.
Blinding	Data collection was not blinded as sample treatment and collection requires prior knowledge of the experiment. Data was first processed in blinded mode, followed by sample/control assignment.

Reporting for specific materials, systems and methods

We require information from authors about some types of materials, experimental systems and methods used in many studies. Here, indicate whether each material, system or method listed is relevant to your study. If you are not sure if a list item applies to your research, read the appropriate section before selecting a response.

Materials & experimental systems

n/a	Involved in the study	
☐	☒	Antibodies
☐	☒	Eukaryotic cell lines
☒	☐	Palaeontology and archaeology
☒	☐	Animals and other organisms
☒	☐	Clinical data
☒	☐	Dual use research of concern

Methods

n/a	Involved in the study	
☒	☐	ChIP-seq
☒	☐	Flow cytometry
☒	☐	MRI-based neuroimaging

Antibodies

Antibodies used	Ms anti-Erk CST 9107 1:1000<br>Rb anti-phospho-Erk CST 9101 1:2000<br>Ms anti-Akt CST 2920 1:1000

	Rb anti-phospho-Akt (S473) CST 9271 1:1000<br>Rb anti-GAPDH CST 5174 1:1000<br>Ms anti-Vinculin Sigma-Aldrich V9131 1:1000<br>Ms anti-Myc Tag CST 2276 1:1000<br>Ms anti-Flag Tag Sigma-Aldrich A8592 1:1000<br>Rb anti-E cadherin Abcam ab40772 1:1000<br>Rb anti-Sec61B Abcam ab15576 1:1000<br>Ms anti-Giantin Abcam ab37266 1:1000<br>Rb anti-pan Ras Millipore 05-1072 1:1000<br>Rb anti-HA Tag CST 3724 1:1000<br>Rb anti-GFP CST 2555 1:1000<br>Rb anti-PAPOLG Thermo Fisher PA5-41901 1:1000<br>Rb anti-MVP Novus NBP1-33560 1:1000<br>Ms anti-MVP Fisher Scientific MS664P1ABX 1:1000<br>Rb anti-VDAC1 Abcam ab15895 1:1000<br>Rb anti-pan VDAC Alomone Labs AVC-001 1:1000<br>Rb anti-Shp2 CST 3397S 1:1000<br>Ms anti-EGFR Abcam ab30 1:1000<br>Rb anti-FGFR3 CST 4574S 1:1000<br>Gt anti-Biotin Vector Labs SP-3000-1 1:1000<br>Rb anti-Biotin Abcam ab53494 1:1000<br>Ms anti-Biotin Abcam ab201341 1:1000<br>Rb anti-KRas Millipore OP24 1:1000<br>Ms anti-HRas Proteintech 18295-1-AP 1:1000<br>Rb anti-NRas Santa Cruz Biotechnology SC- 1:100031<br>Rb anti-Hsp60 CST 12165S 1:1000<br>Ms anti-Hsp60 Abcam ab59457 1:1000<br>Duolink® In Situ PLA® Probe Anti-Goat PLUS Millipore Sigma DUO92003 1:200<br>Duolink® In Situ PLA® Probe Anti-Goat MINUS Millipore Sigma DUO92006 1:200<br>Duolink® In Situ PLA® Probe Anti-Rabbit PLUS Millipore Sigma DUO92002 1:200<br>Duolink® In Situ PLA® Probe Anti-Rabbit MINUS Millipore Sigma DUO92005 1:200<br>Duolink® In Situ PLA® Probe Anti-Mouse PLUS Millipore Sigma DUO92001 1:200<br>Duolink® In Situ PLA® Probe Anti-Mouse MINUS Millipore Sigma DUO92004 1:200<br>680 RD anti-Ms LICOR 26-68071 1:10000<br>680 RD anti-Rb LICOR 926-68070 1:10000<br>800 CW anti-Ms LICOR 926-32210 1:10000<br>800 CW anti-Rb LICOR 926-32211 1:10000<br>AF488 anti-Ms Thermo Fisher A11029 1:200<br>AF568 anti-Rb Thermo Fisher A11036 1:200<br>AF488 anti-Rb Thermo Fisher A11034 1:200<br>AF750 anti-Ms Thermo Fisher A21037 1:200
Validation	Validation of all antibodies are provided on manufacturer's websites for specific application (immunostaining, immunoblotting). All of these antibodies have been used in previous literature as well. Example data is shown on manufacturer's website (e.g. target protein KO).<br>For all CST primary antibodies listed here, antibodies are validated in gene KO cells siRNA KD cells, heterzygous KO cells, different cell lines, and in other antibody applications such as competitive ELISA, peptide dot blots, peptide blocking, or protein arrays.<br>For all Sigma-Aldrich, Thermo Fisher, Vector Labs, Proteintech, and Millipore antibodies listed here, antibodies underwent enhanced validation efforts which include validation in gene KO cells, gene overexpression cells, and orthogonal assays (e.g. RNAseq correlation, Immunocapture-MS).<br>Abcam and Novus antibodies were validated in KO cells.<br>Santa Cruz Biotechnology and Alomone Labs antibodies were validated in siRNA KD cells.

Eukaryotic cell lines

Policy information about cell lines and Sex and Gender in Research

Cell line source(s)	Lemo21(DE3) NEB C2528J<br>MIA PaCa-2 ATCC CRL-1420<br>H358 ATCC CRL-5807<br>MEF WT NCI<br>SW1573 ATCC CRL-2170
Authentication	Cell lines were authenticated by provider.<br>MEF cells were sequenced.<br>All ATCC cell lines have undergone STR profiling.
Mycoplasma contamination	All cell lines were tested every 2 months for mycoplasma contamination via PCR test. None of these cell lines were positive for mycoplasma.
Commonly misidentified lines<br>(See ICLAC register)	None in this study.
