# Supplementary figures and images for: Single-cell sensor analyses reveal signaling programs enabling Ras-G12C drug resistance
Source: Nat Chem Biol. 2024 Aug 5;21(1):47–58. doi: 10.1038/s41589-024-01684-4 (PMC11666463; doi:10.1038/s41589-024-01684-4)

Figure 2e

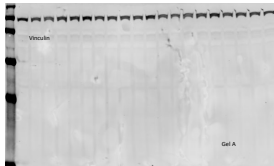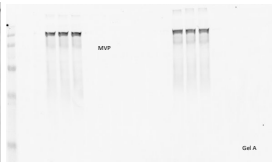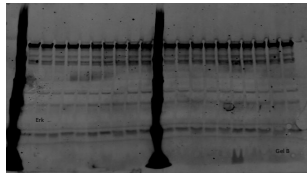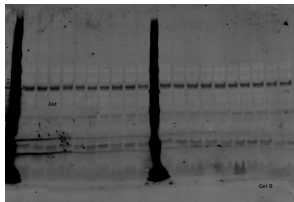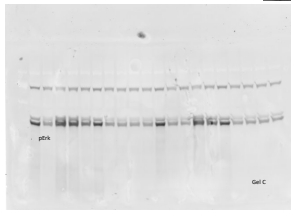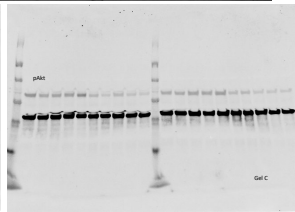

Supplement: Supplementary file 4 — Source data and unprocessed western blots. [file 41589_2024_1684_MOESM4_ESM.zip › Fig 2/Gels for Fig 2.pdf]

Figure 4e

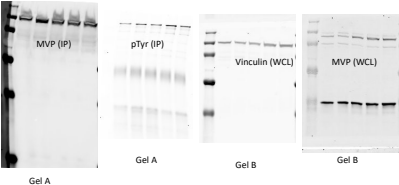

Figure 4f

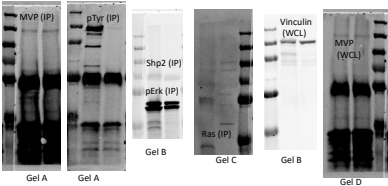

Supplement: Supplementary file 6 — Source data and unprocessed western blots. [file 41589_2024_1684_MOESM6_ESM.zip › Fig 4/Gels for Fig 4.pdf]

# Figure 6a

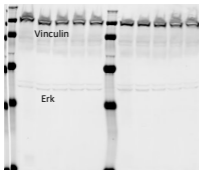

Gel A

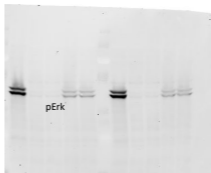

Gel B

# Figure 6c

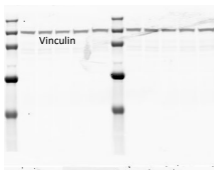

Gel A

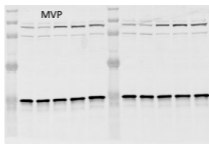

Gel A

Supplement: Supplementary file 8 — Source data and unprocessed western blots. [file 41589_2024_1684_MOESM8_ESM.zip › Fig 6/Gels for Fig 6.pdf]

# Extended Data Figure 1a

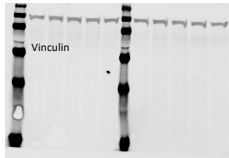

Gel A

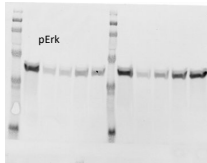

Gel A

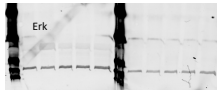

Gel B

Supplement: Supplementary file 9 — Source data and unprocessed western blots. [file 41589_2024_1684_MOESM9_ESM.zip › ED Fig 1/Gels for EDFig 1.pdf]

# Extended Data Figure 3h

# Extended Data Figure 3k

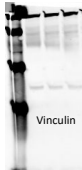

Gel A

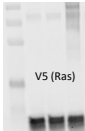

Gel B

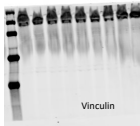

Gel A

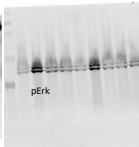

Gel A

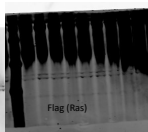

Gel A

Supplement: Supplementary file 11 — Source data and unprocessed western blots. [file 41589_2024_1684_MOESM11_ESM.zip › ED Fig 3/Gels for EDFig 3.pdf]

# Extended Data Figure 4b

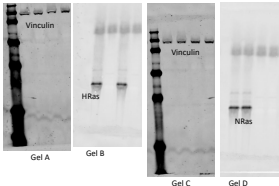

Supplement: Supplementary file 12 — Source data and unprocessed western blots. [file 41589_2024_1684_MOESM12_ESM.zip › ED Fig 4/Gels for EDFig 4.pdf]

# Extended Data

## Figure 5f

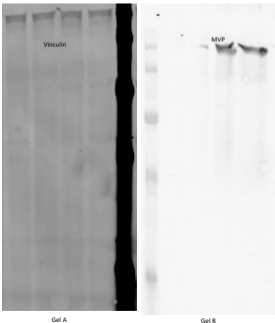

Supplement: Supplementary file 13 — Source data and unprocessed western blots. [file 41589_2024_1684_MOESM13_ESM.zip › ED Fig 5/Gels for EDFig 5.pdf]

# Extended Data Figure 6b

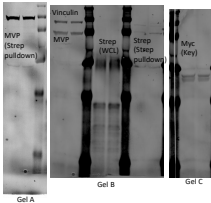

Supplement: Supplementary file 14 — Source data and unprocessed western blots. [file 41589_2024_1684_MOESM14_ESM.zip › ED Fig 6/Gels for EDFig 6.pdf]
